# Emerging Targets, Novel Directions, and Innovative Approaches in Thrombosis Therapy

**DOI:** 10.14336/AD.2024.1688

**Published:** 2025-03-11

**Authors:** Weiyue Zhang, Baoqing Pei, Yifan Zhou, Hui Li, Wei Ma, Bing Zhou, Chen Zhou, Huimin Jiang, Xunming Ji

**Affiliations:** ^1^Beijing Advanced Innovation Center for Big Data-Based Precision Medicine, School of Biological Science and Medical Engineering, Beihang University, Beijing, 100191, China.; ^2^Beijing Institute of Brain Disorders, Laboratory of Brain Disorders, Ministry of Science and Technology, Collaborative Innovation Center for Brain Disorders, Beijing Advanced Innovation Center for Big Data-Based Precision Medicine, Capital Medical University, Beijing, 100069, China.; ^3^Department of Neurology, Xuanwu Hospital, Capital Medical University, Beijing, 100053, China.

**Keywords:** Thrombosis, Platelets, Coagulation pathway, Inflammation, Nanomedicine

## Abstract

In clinical practice, antiplatelet, anticoagulant and fibrinolytic drugs are the mainstay of thrombosis treatment, but their potential bleeding side effects limit their widespread use. Therefore, modifying these existing drugs or developing new therapies that mitigate bleeding risks while maintaining their efficacy and utilization is necessary. Since the critical role of platelets in thrombosis is closely related to their cell surface receptors, intracellular signaling pathways and metabolism, current research focuses on these three major classes of platelet targets to develop new antithrombotic drugs. The coagulation cascade has always been the main target of anticoagulant drugs, but since the role of molecules of the contact system is more critical in thrombosis than in hemostasis, molecules targeting the contact system, such as FXIa and FXIIa, have become the main direction of anticoagulant drug research at present. Moreover, since the inflammatory response has been found to be significantly associated with thrombosis in recent years, the development of drugs that target inflammatory pathways, such as inflammasome, has also become a hot topic. This article provides a detailed description of these targets or drug formulations that are currently being investigated, including their mode of action and antithrombotic efficiency, and also points out their existing shortcomings. Moreover, antithrombotic nanomedicines can achieve precise release of drugs, which can greatly improve the thrombolytic efficiency and reduce side effects. In conclusion, this review focuses on summarizing the current new targets and new methods of antithrombotic drug research, hoping to provide a little reference for future related research.

## Introduction

Cardiovascular and cerebrovascular diseases are the global leading causes of disability and death [1], with their incidence steadily rising and increasingly affecting younger populations [2]. The formation and spread of blood clots are key factors in the development and progression of these diseases. When blood flows through vessels, damage to the inner lining or dysfunction of endothelial cells exposes subendothelial collagen fibers, triggering platelet activation and aggregation. This aggregation forms platelet clumps, and fibrinogen is then converted into fibrin. The platelets adhere to the vessel walls, creating a structure known as a platelet plug. Fibrin further ensnares white and red blood cells, gradually forming a clot. This process is known as thrombosis [3].

This article summarizes the research status of new drug targets, as well as novel drugs and formulations, based on the roles of platelets, coagulation factors, and inflammation in thrombosis. Additionally, we explore the advancements in nanomedicine delivery for the treatment of thrombosis. Importantly, this article points out the advantages and disadvantages of the new drugs currently in development and provides ideas and directions for further research.

## 1. Current status of thrombosis research

### 1.1 Classification and risk factors

Thrombosis is classified based on its location as either arterial thrombosis (AT) or venous thrombosis (VT). AT is associated with ischemic stroke, acute coronary syndrome (ACS), atrial fibrillation (AF), and transient ischemic attack (TIA) [4]. In contrast, VT can give rise to diseases like pulmonary embolism (PE), deep vein thrombosis (DVT), and cerebral venous thrombosis (CVT) [4].

Rudolf Virchow identified three key elements of thrombosis: blood stasis, endothelial injury, and hypercoagulable state. Blood stasis predominantly affects the venous system, leading to VT. Key risk factors include prolonged bed rest, post-surgical recovery, pregnancy, and tumors, which slow down the flow of blood in the veins and elevate the risk of clot formation [5, 6]. Endothelial injury typically occurs in the arterial system. Smoking, hypertension, atherosclerosis, and diabetes damage the arterial lining, activating the coagulation process, which then induces AT [7-9]. Hypercoagulable state can heighten the risk of both AT and VT. Obesity, cancer, pregnancy, and aging are key contributors to this condition, making blood more prone to clotting by increasing coagulation factors and reducing anticoagulants [7-10]. In summary, different risk factors elevate the risk of VT, AT, or both by acting on one or more of the three key elements of thrombosis.

## 1.2 Mechanisms of thrombosis

### 1.2.1 Blood component abnormalities

Platelet activation is an integral part of the process of thrombosis. Arterial blood flow has a fast flow and high pressure, making atherosclerotic plaques more susceptible to rupture under high-flow conditions. When an atherosclerotic plaque ruptures, the endothelium is damaged, exposing subendothelial collagen. Von Willebrand factor (vWF) is a multimeric glycoprotein synthesized and secreted by vascular endothelial cells and megakaryocytes. It binds to the collagen surface, exposing its platelet binding site [11]. Platelets adhere to collagen by binding the glycoprotein (GP) Ⅰb-V-Ⅸ complex present on their surface to vWF [11]. Additionally, the platelet receptors α2β1 and GPVI play a key role in promoting platelet aggregation and activation at the collagen interface [12]. This adhesion phase triggers platelet activation and cytoskeletal reorganization. Activated platelets release aggregation mediators such as thromboxane A2 (TxA2), ADP, and serotonin, and produce thrombin, which recruits other circulating platelets [13]. Ultimately, platelets aggregate through the activation of their GPIIb/IIIa integrins, forming 3D clot structures [14, 15]. Slightly different from AT, in VT, HMGB1 promotes the recruitment of monocytes, which in turn oxidize HMGB1. This oxidation process enhances its prothrombotic activity and inducing platelet aggregation. HMGB1 also stimulates the formation of neutrophil extracellular traps (NETs), which is a network of chromatin-based backbones incorporating a variety of antimicrobial proteins and enzymes that are released by neutrophils in response to stimulation, further driving the development of VT [16]. Additionally, platelets can facilitate leukocyte recruitment via their GPIbα receptors, P-selectin, and early inflammatory cells, creating synergies that promote neutrophil recruitment. Neutrophils, in turn, rely on coagulation processes to advance VT progression [17].

In AT, when platelets are swiftly activated and adhere to the injury site through vWF, interactions between coagulation factor VII (FVII) and tissue factor (TF, a transmembrane glycoprotein, mainly expressed by fibroblasts and other cells in the vascular epithelium, which acts as a receptor for coagulation FVII and can initiate the coagulation cascade) are initiated, which culminates in the activation of FX, the generation of thrombin, and the promotion of fibrin formation. In contrast, VT depends primarily on the endogenous coagulation pathway and usually occurs in the presence of slow or stagnant blood flow. Blood stasis can cause local hypoxia, damage endothelial cells, and impair their anticoagulant function. At this time, the activation of FVIII, FIX, FXI, and FXII promotes thrombin production, fibrin formation, and thrombus stabilization [18, 19]. In addition, venous valve dysfunction can cause blood regurgitation and local stasis, further increasing the risk of VT.

### 1.2.2 Disruption of blood vessels

The arterial wall is thick and contains abundant smooth muscle and elastic fibers, allowing it to withstand high pressure. However, prolonged exposure to a high-pressure environment can lead to endothelial cell damage. Once endothelial cells are damaged, subendothelial collagen and TF are exposed, promoting platelet adhesion and activating coagulation factors, which leads to thrombus formation. Additionally, during atherosclerosis, the arterial wall becomes stiffer, which increases the chance of plaque breakup and further contributes to AT.

In contrast, veins have a simpler structure with thinner, less elastic walls and lower smooth muscle content, making them more vulnerable to damage from external forces or pressure. This structural weakness makes venous endothelial cells more prone to rupture even from minor injuries, exposing the underlying matrix, which induces platelet adhesion and activates the coagulation mechanism, leading to thrombus formation. Furthermore, veins are more likely to deform under prolonged stress, such as in cases of tumors or pregnancy, which further increases the risk of VT [5, 6].

### 1.2.3 Systemic disease

Thrombosis is especially associated with the progression of systemic diseases and inflammation. Specifically, there is a complex interaction between innate immunity, platelet activation and the coagulation system that leads to thrombosis, which is called immunothrombosis. Excessive activation of immunothrombosis can lead to thromboinflammation, creating a vicious cycle in which platelets and innate immune cells are activated, releasing components of the complement system, which then tiggers the coagulation cascade, and further worsens thrombosis [20].

Activated interactions between platelets and innate immune cells are mediated through coagulation and complement systems. Additionally, macrophage-mediated pyroptosis significantly activates the coagulation system [21]. Platelets recruit and activate neutrophils by releasing soluble mediators such as CCL5, CXCL4, CXCL5, and MIF, along with adhesion molecules [22, 23]. Once activated, neutrophils not only accumulate at the site of thrombosis but also contribute to its spread through a process known as NETosis. NETs form a key link between thrombosis and inflammation. The extracellular DNA structure of NETs, when binding to vWF, enhances platelet adhesion and aggregation [24, 25]. The histones within NETs further stimulate platelet aggregation, activate platelets, and promote clot formation. In addition, the negative charge on the surface of NET DNA facilitates the combination and activation of FXII, triggering the intrinsic coagulation pathway. Moreover, neutrophil elastases found in NETs can degrade TF pathway inhibitors, leading to increased thrombin generation [26]. This thrombin, in turn, stimulates further platelet production and activation, creating a self-perpetuating cycle that intensifies both inflammation and thrombosis. Inflammasome activation further induces gasdermin D (GSDMD)-dependent pyroptosis in macrophages, releasing TF-containing microparticles [21]. TFs are key activators of exogenous coagulation pathways and are strongly prothrombotic.

**Figure 1.**
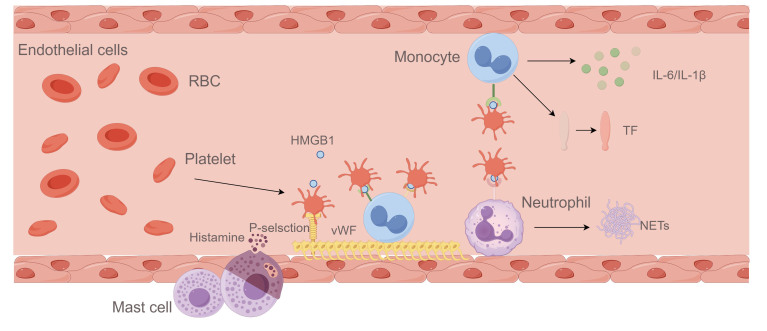
**Immune response and venous thrombosis**. Reduced blood flow in veins triggers thrombosis via sterile inflammation and the coagulation cascade. Sluggish flow activates perivascular mast cells to release histamine and endothelial cells to mobilize P-selectin and vWF, recruiting platelets and immune cells like monocytes and neutrophils. Platelets release HMGB1, aggregating monocytes and neutrophils. Neutrophils release NETs, while monocytes secrete IL-6, IL-1β, and TF, which binds FXII on NETs to initiate coagulation.

In VT, the role of inflammatory response in thrombosis has its own particularity. Normally, the vessel wall remains intact, the subendothelial collagen or TF is not exposed, and the reduced venous blood flow triggers an immune response [27]. Decreased shear stress activates inflammatory NF-κB pathways, increases expression of adhesion molecules and promotes leukocyte recruitment [28]. Moreover, slow or stagnant venous blood flow can lead to endothelial hypoxia. In response, hypoxia-inducible factor-1α (HIF-1α) stimulates NLRP3 inflammasome expression in endothelial cells, resulting in IL-1β secretion and an inflammatory response [29]. Additionally, reduced flow activates endothelial cells by releasing mast cell mediators (e.g. histamine), and exposes vWF, which initiates a complex interaction among platelets, neutrophils, and monocytes, as described earlier [25]. And the role of inflammation in VT is depicted in detail in Figure 1. In summary, it plays an important role in the pathophysiology of VT, yet remains an unresolved challenge in its treatment [30].

### 1.3 Formation process and composition

**Figure 2.**
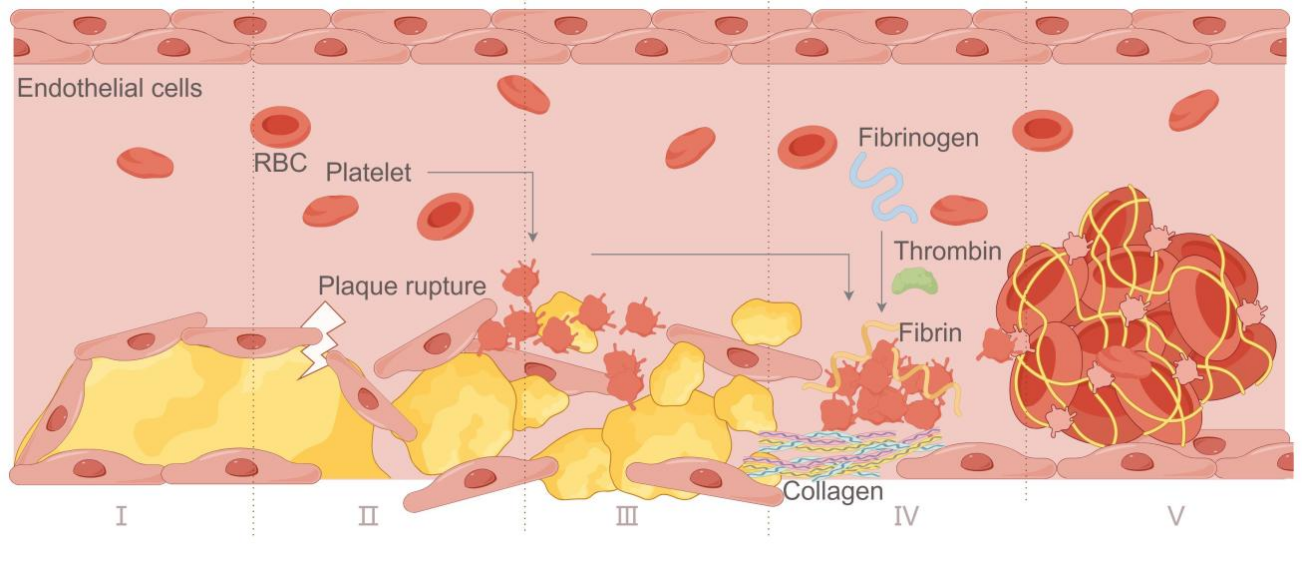
**Arterial thrombosis process**. Ⅰ. Atherosclerotic plaque formation; Ⅱ. Rupture of atherosclerotic plaque; Ⅲ. Platelet activation; Ⅳ. Thrombosis; Ⅴ. Vascular obstruction

The mechanism of AT and VT is different, which directly determines the differences in their formation process and composition. Figure 2 presents in detail the formation process of AT. AT is closely associated with arteriosclerosis, a chronic inflammatory process which causes cholesterol and fat to build up in the artery wall and form a plaque [31]. With the enlargement of the plaque, the arterial lumen gradually narrows, and blood flow is restricted. When plaque rupture or ulceration occurs, the exposed lipid core and TF trigger platelet activation and coagulation response, marking the onset of thrombosis. Platelets rapidly accumulate on the damaged endothelial surface and bind to the endothelium via vWF, forming preliminary platelet emboli. The formation of thrombin then accelerates the formation of fibrin, and the network of fibrin holds platelets, erythrocytes and leucocytes together to form a stable thrombus. As the thrombus grows, it can completely block the lumen, interrupting blood flow and causing acute ischaemic events such as MI or stroke [32]. Arterial thrombi, which can usually be divided into white thrombi and mixed thrombi. The former is composed mainly of platelets and fibrin, and the latter is mixed with red and white blood cells.

**Figure 3.**
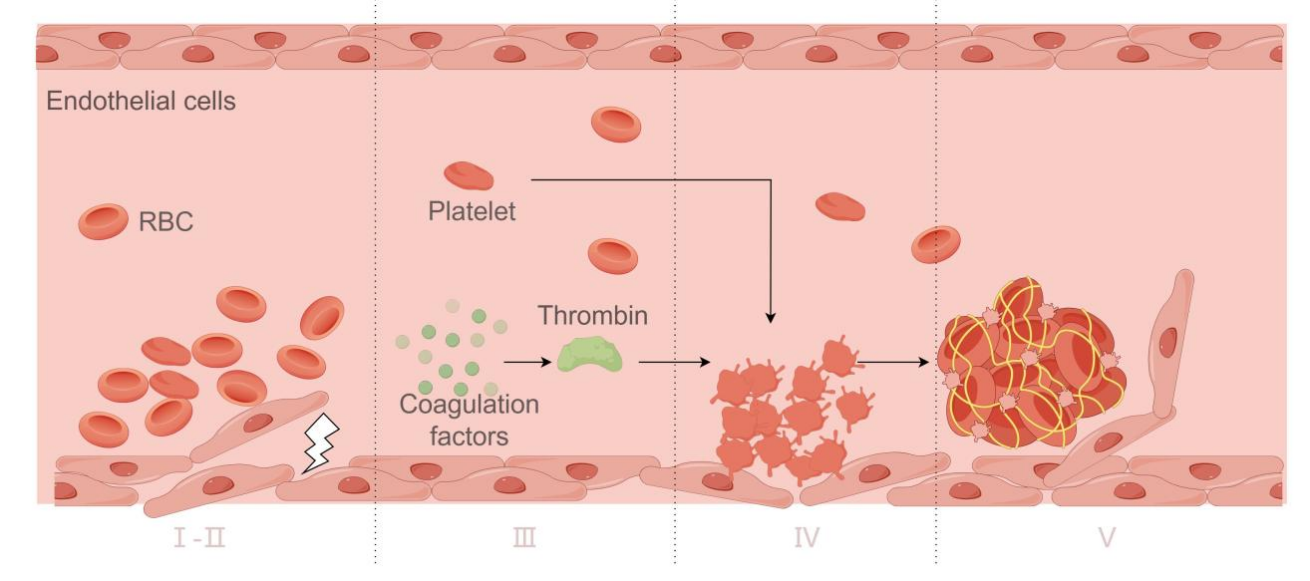
**Venous thrombosis process**. Ⅰ. Blood flow stasis; Ⅱ. Vessel wall damage; Ⅲ. Coagulation factor activation; Ⅳ. Platelet aggregation; Ⅴ. Fibrin formation

VT formation is closely related to blood stasis, vascular wall injury and hypercoagulable state. Figure 3 presents in detail the formation process of VT. First, venous stasis is a key factor in VT, which usually occurs during prolonged bed rest, sitting, or venous valve insufficiency, resulting in slow local blood flow and making coagulation factors and platelets more likely to accumulate in restricted areas. Second, when venous endothelial cells are damaged, for example due to trauma, surgery, or disease, the exposed matrix components such as collagen fibers activate platelets and coagulation factors, thereby initiating the coagulation reaction and marking the onset of thrombosis [33]. During this process, coagulation factors are activated, especially FVIII, FIX, FXI, and FXII in the endogenous coagulation pathway, which generate thrombin and further contribute to fibrin formation. The fibrin network holds platelets, erythrocytes and leucocytes together to gradually form a stable thrombus. With the enlargement of the thrombus, it may completely block the vein, affect blood return, and even lead to serious complications such as PE. The major elements of venous thrombus are red blood cells and fibrin, which usually form red thrombi, rich in erythrocytes, accompanied by fewer platelets. The formation process and components of VT and AT respectively determine their specific pathological manifestations in vascular lesions.

### 1.4 Treatment methods

Currently, the primary treatments for thrombosis include surgical interventions and drug therapies, with approaches varying between AT and VT. For AT, thrombectomy is typically the first-line treatment, provided it is performed within the appropriate time window [34], followed by drug therapy. In contrast, VT is generally managed with drug therapy as the initial treatment, with thrombectomy reserved for more severe cases.

Clinically, drugs used for treating thrombosis are primarily categorized into three groups: antiplatelet agents (Supplementary table 1), anticoagulants (Supplementary table 2), and thrombolytics. Antiplatelet agents include aspirin, clopidogrel and ticagrelor are the main drugs used for treating AT [35]. These drugs have the effect of blocking platelet aggregation, thereby reducing the risk of thrombosis. The main anticoagulants used to prevent and treat VT and thromboembolic events associated with AF are heparin, warfarin and the newer direct oral anticoagulants (DOACs). These drugs prevent the formation and extension of clots by inhibiting coagulation factors.

## 2. New targets for the treatment of thrombosis

### 2.1 New targets for antiplatelet therapy

**Figure 4.**
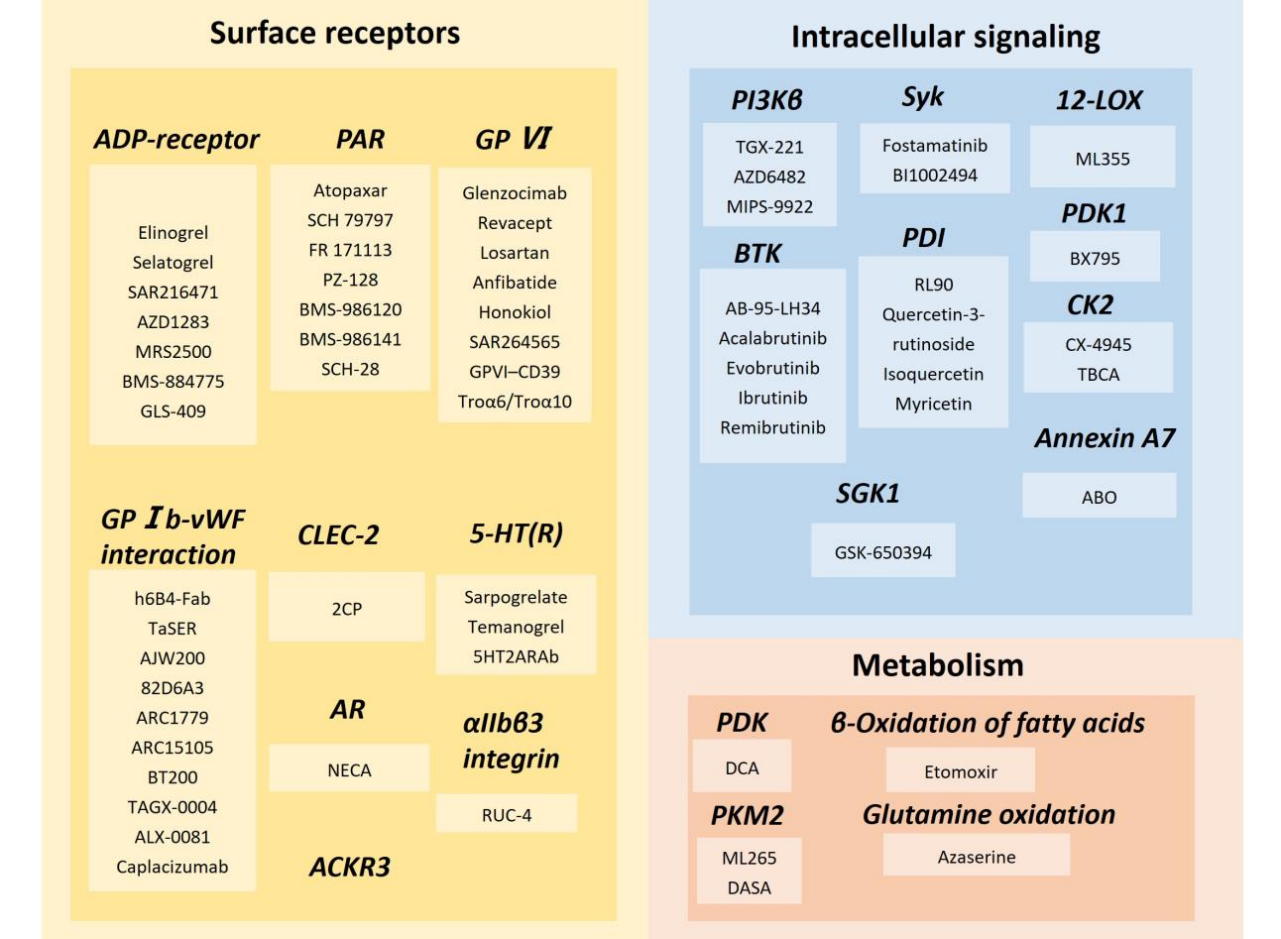
**New targets for antiplatelet therapy**. According to the type of target, it can be divided into cell surface receptors, intracellular signaling molecules and metabolic pathway-related molecules.

Platelets play an important role in thrombosis. When the vascular endothelium is damaged, platelets attach rapidly to the exposed collagen fibers at the site of injury. After adhesion, platelets are activated, undergo morphological changes, extend pseudopodia, and secrete a variety of substances including adenosine diphosphate (ADP) and TxA2. ADP and TxA2 can attract additional platelets to the injured site, form a platelet thrombus, and initially block the vascular rupture. Furthermore, platelets expose a phospholipid surface to clotting factors, promoting their activation, which converts prothrombin to thrombin, which converts fibrinogen to fibrin, reinforcing the platelet clot and ultimately forming a solid thrombus. Antiplatelet antithrombotic agents or reagents are also under investigation, and specific targets and drugs or agents are summarized in Figure 4 and Supplementary Table 3.

### 2.1.1 Antiplatelet drugs targeting surface receptors

Targeting ADP receptors: ADP-induced platelet aggregation is mediated by two different platelet surface G protein-coupled receptors (GPCRs): P2Y12 and P2Y1 [36]. When ADP binds to these receptors, it activates different signaling pathways: P2Y1 stimulates phospholipase Cβ (PLCβ), while P2Y12 activates phosphatidylinositol 3-kinase (PI3K). These pathways lead to changes in platelet shape and promote subsequent aggregation [37].

*Elinogrel:* Elinogrel is a P2Y12 antagonist with a mechanism similar to that of clopidogrel and ticagrelor but is unique in that it can be administered intravenously and orally, providing the flexibility of rapid onset and sustained antiplatelet effects. In patients undergoing non-urgent PCI, a single oral dose of 100 mg plus an intravenous dose of 120 mg produced a faster and more potent antiplatelet effect than standard clopidogrel in patients already on dual antiplatelet therapy (NCT00751231) [38]. However, the termination of its development and the lack of large-scale clinical trial data limit its clinical significance. Future studies are needed to further verify its efficacy and safety and clarify its position in antiplatelet therapy.

*Selatogrel (ACT-246475):* Selatogrel is a novel reversible binding P2Y12 inhibitor that is unique in that it is administered by subcutaneous injection and can be rapidly administered in the prehospital or home setting, making it suitable for emergency self-rescue in patients with AMI [39, 40]. A single dose of selatogrel produced rapid and sustained platelet inhibition for more than 8 hours in a clinical trial of patients with chronic coronary syndrome (CCS) (NCT03384966) [41]. However, its clinical significance still needs to be verified by further large-scale clinical trials. Future research is needed to clarify its position in the emergency treatment of AMI and optimize the patient’s use strategy.

*SAR216471:* SAR216471 is a potent, highly selective, and reversible antagonist of the P2Y12 receptor [42]. It aims to lower levels of low-density lipoprotein cholesterol (LDL-C), thereby reducing the risk of cardiovascular disease. Using human platelet-rich plasma, SAR216471 suppressed ADP-induced platelet aggregation at an IC50 of 108nM. Importantly, this effect can be translated into antithrombotic activities in vivo with low bleeding risk [43]. Despite the favorable short-term safety profile, the potential risks of long-term use need to be further studied. This, coupled with its high price, may limit its use in low-income patients. Future studies should focus on its long-term efficacy and economic benefits to better evaluate its clinical application value.

*AZD1283:* AZD1283 is a novel P2Y12 receptor antagonist. A series of novel bicyclic pyridine derivatives as potential P2Y12 antagonists were designed and synthesised on the basis of AZD1283. Among these, compound 58l exhibited strong in vitro platelet aggregation inhibition and showed antithrombotic activity in a rat model of FeCl_3_ injury [44]. AZD1283 failed to demonstrate significant advantages over existing drugs, and there is a lack of sufficient clinical data to support its widespread use. Future studies should focus on its potential value in specific patient groups, such as those intolerants to existing drugs, and further assess its safety and efficacy.

*MRS2500:* MRS2500, a nucleotide analogue, is a potent and selective antagonist of the P2Y1 receptor. In mice, intravenous administration of MRS2500 protected against systemic thromboembolism induced by a mixture of collagen and epinephrine [45]. However, MRS2500 is mainly used in laboratory research and has not yet entered the clinical trial stage, and its safety and efficacy have not been verified in humans. Future studies could explore the combination of MRS2500 with other antiplatelet drugs or develop novel antithrombotic drugs based on P2Y1 receptor inhibition.

*BMS-884775:* Unlike previous adenine nucleotide P2Y1 antagonists such as MRS2500, BMS-884775 is orally administered [46, 47]. Testing in a efficacy/bleeding model in rabbits revealed that BMS-884775 had similar antithrombotic efficacy to prasugrel, but with a reduced risk of bleeding [48]. However, its progress in clinical development progress is limited, and its safety and efficacy still need to be further verified.

*GLS-409:* GLS-409, a diadenosine tetraphosphate (Ap4A) derivative, is a synergistic antiplatelet agent that inhibits both P2Y1 and P2Y12 receptors. Dual inhibition may reduce the patient's risk of developing drug resistance as compared with a single-receptor antagonist. GLS-409 rapidly improved coronary patency in a canine model of platelet-induced thrombosis [49, 50]. Further testing showed that low dose GLS-409 was effective in inhibiting recurrent coronary thrombosis in vivo without prolonging bleeding time [51]. However, the clinical development of GLS-409 has not yet entered an advanced stage, and large-scale clinical trial data are lacking, and its safety and efficacy have not been fully verified in humans. Future studies should focus on its safety and efficacy, especially in the assessment of bleeding risk, and explore its value in specific patient groups, such as those who are intolerant to existing drugs.

**Targeting Protease-activated receptor (PAR)**: Thrombin interaction with PAR1 and PAR4 results in cleavage of platelets' extracellular domains [36].

*Atopaxar (E5555):* Atopaxar is a reversible PAR1 antagonist that disrupts platelet signaling. In ACS patients, atopaxar significantly reduced early ischemia (NCT00548587) [52]. In a dose-ranging study in CAD patients, atopaxar effectively suppressed platelets and reduced ischaemic events, but caused a modest increase in total bleeding compared to placebo (NCT00312052) [53]. However, atopaxar's clinical development was halted at a later stage, in part because it was not significantly more effective than existing agents and was associated with a higher risk of bleeding.

*SCH79797:* SCH79797 is a selective antagonist of the PAR1 receptor. In a rat model of myocardial ischaemia-reperfusion (I/R) injury, a single dose of SCH79797 administered before or during the ischaemic event provided immediate cardiac protection [54]. At present, the research is mainly limited to animal models and in vitro experiments, and there is a lack of large-scale clinical trial data. Its safety and efficacy still need to be verified, and its mechanism of action may limit its application in complex diseases.

*FR171113:* FR171113 is a selective PAR1 receptor antagonist that has been used to study the function of the PAR1 receptor. In guinea-pig carotid artery thrombosis model, treatment with 1.0 mg/kg FR171113 did not prolong the bleeding time. Importantly, even at 32 mg/kg, FR171113 had no prolongation of bleeding time [55]. However, the neuroprotective effect of FR171113 still needs more experimental support, and its long-term safety is unknown. Furthermore, since FR171113 may inhibit 5-HT2A receptors which play a role in a variety of physiological processes, long-term use of FR171113 may lead to metabolic disturbances, cardiovascular problems, or other unknown side effects.

*PZ-128:* PZ-128 targets the cytoplasmic domain of the PAR1 receptor, acting as a rapid and reversible antiplatelet agent [56, 57]. A phase II study (NCT02561000) found PZ-128 safe and well tolerated when added to standard antiplatelet therapy, possibly reducing periprocedural myonecrosis in patients undergoing cardiac catheterisation for PCI [58]. However, the inhibition of PAR1 alone may have limited effect and may induce compensatory activation of other pathways. The long-term effects of its use are unclear and may pose a risk of bleeding or other unknown side effects, requiring further study.

*BMS-986120:* BMS-986120 is a potent and reversible small molecule antagonist of the PAR4 receptor. In a phase I trial (NCT02439190), oral BMS-986120 (20mg) reduced total thrombus area (μm^2^/mm) in vitro by 29.2% at higher shear rates, but had no effect on thrombus formation at lower shear rates [59]. However, its long-term safety needs to be further verified, especially in high-risk patients (such as the elderly or patients with renal insufficiency). BMS-986120 may synergize with traditional antiplatelet agents, such as aspirin and P2Y12 inhibitors, to provide greater antithrombotic efficacy. However, the combination therapy may increase the risk of bleeding, and the optimal dose and treatment regimen require further investigation.

*BMS-986141:* BMS-986141 is a small molecule PAR4 antagonist with improved oral bioavailability and clinical efficacy over BMS-986120 (NCT02341638) [60]. However, no significant additional benefit was found when it was added to aspirin, although it further reduced the risk of recurrent stroke [60]. The final confirmation of its clinical significance still depends on the results of more large-scale and long-term clinical studies.

***SCH-28**:*SCH-28 is a small-molecule, synthetic, non-anticoagulant heparin analogue that inhibits thrombin-induced PAR4 activation. In an in vitro model of thrombosis, SCH-28 was effective in reducing thrombosis under conditions of total arterial blood flow [61]. However, as an early research drug, the problems of drug metabolism, toxicity and bioavailability have not been solved.

Targeting GPⅥ: GPⅥ, a key collagen signalling receptor on platelets, is important in AT and inflammation and contributes to the stabilization of platelet thrombi, but its role in hemostasis appears to be minimal [62].

*Glenzocimab (ACT017):* Glenzocimab is a humanised monoclonal antigen-binding fragment (Fab). It is directed against human platelet GPⅥ. In a phase II study (NCT03803007) in patients with acute stroke, the combination of glenzocimab (1000mg) with alteplase, with or without mechanical removal of the clot, was associated with a reduction in serious adverse events, intracranial bleeding and mortality [63]. The application prospect of glenzocimab in acute ischemic stroke and cardiovascular diseases is worth looking forward to, but its clinical significance still needs to be supported by more large-scale clinical trials.

*Revacept:* Revacept, an antagonist of collagen-mediated platelet activation, revacept is a soluble dimeric GPVI receptor fusion protein. In a mouse ischaemic stroke model, 1 mg/kg IV revacept significantly improved functional outcome, infarct size and cerebral oedema right before I/R [64]. Revacept was shown to inhibit collagen-induced platelet activation in a dose-dependent manner without affecting hemostasis in a phase Ⅰ study (NCT01042964) [65]. Although revacept has shown promising efficacy in multiple studies, its effect may be closely related to the dose. For example, in patients with symptomatic carotid stenosis, the 120 mg dose was more effective than the 40 mg dose [66]. Most of the current clinical trials are short-term follow-up, and further studies are needed to evaluate the long-term efficacy and safety of revacept.

*Losartan (DuP-753):* Losartan, a commonly used antihypertensive drug, was also shown to be effective in inhibiting GPⅥ-mediated aggregation of blood platelets. EXP3179, the active metabolite of losartan, inhibited platelet aggregation by human plaque material from atherosclerotic lesions in vitro and reduced platelet adhesion in mice after acoustic vascular injury in vivo [67]. However, it may cause hyperkalemia, renal impairment, or hypotension, especially in elderly patients or those with renal insufficiency. In addition, it may be harmful to the fetus during pregnancy and should be avoided.

*Anfibatide:* Anfibatide is a peptide derived from venom that selectively inhibits platelet aggregation mediated by GPⅥ. In a model of transient middle cerebral artery occlusion (MCAO), treatment with anfibatide significantly reduced ischaemic lesions in a dose-related manner and significantly decreased the incidence of intracerebral haemorrhage and the duration of tail bleeding compared to tirofiban [68]. However, the complexity of its mechanism of action and its long-term efficacy still needs further investigation. Future research should focus on optimizing its dosage, expanding the range of indications, and exploring the possibility of combination therapy to fully realize its clinical value.

*Honokiol:* Honokiol is a natural small-molecule compound extracted from the bark of Magnolia officinalis. In a rat model of electric current induced carotid artery thrombosis, honokiol significantly prolonged thrombus obstruction time in a dose-related manner. Its primary antithrombotic mechanisms may involve endothelial cell protection and stimulating PGI2 release [69]. However, studies on cardiovascular protection are mainly limited to animal models, and there is a lack of clinical trial data to support its cardiovascular protection.

*SAR264565:* SAR264565 is highly selective for GPⅥ and completely inhibits the platelet aggregation induced by collagen. SAR264565 dose-dependently inhibited platelet adhesion and fibrin formation on the collagen surface under arterial shear using the Sakariassen in vitro thrombosis perfusion chamber model [70]. Its application in ACS and stroke prevention is worth looking forward to, but its clinical significance still needs to be supported by more large-scale clinical trials. Future studies should focus on its long-term safety, efficacy optimization, and application value in different clinical scenarios.

*GPVI-CD39:* GPVI-CD39 is a novel bifunctional fusion protein that combines the targeting of GPVI with the enzymatic activity of CD39, aiming to treat thrombotic diseases by inhibiting platelet activity and promoting thrombolysis. While GPⅥ-Fc is a very powerful inhibitor of platelet aggregation caused by agglutination of atherosclerotic plaques at high shear flow rates, its efficacy is reduced at low shear flow rates. Fusion of the dimeric soluble form of GP VI with the ADP hydrolase CD39 enhanced antithrombotic effects in a FeCl_3_-induced AT mouse model [71, 72]. Current research is mainly limited to animal models and in vitro experiments, and there is a lack of clinical trial data to support its efficacy and safety.

*Troα6/Troα10:* Troα6 and Troα10 are derived from Trowaglerix. They specifically inhibit platelet aggregation induced by collagen by interfering with the receptor for platelet GPⅥ. Intravenous administration of Troα6 (30 mg/kg) or Troα10 (10 mg/kg) significantly lengthened the occlusive time of thrombus formation in irradiated mesenteric veins of sodium fluorescein-pretreated mice. Importantly, no bleeding side effects were observed in the mouse tail transection model of the drug [73]. The structures of Troα6 and Troα10 may still need to be further optimized to improve their stability and efficacy while reducing possible side effects. For example, improving its specificity or binding ability through molecular design could further enhance its therapeutic efficacy.

**Targeting the GPⅠb-vWF interaction**: GPⅠbα is a key member of the GPⅠb-Ⅸ-Ⅴ complex, which binds to vWF and is central to the initiation of thrombosis in arterial shear. vWF acts as a bridge between subendothelial collagen and platelets, exposed following vascular damage, and also acts as a carrier for FⅧ, protecting it from proteolytic degradation in the circulation.

*h6B4-Fab:* The Fab fragment of 6B4 is a mouse monoclonal antibody that targets platelet GPⅠbα and blocks the binding of vWF. In a study involving baboons, a single intravenous injection of a humanised anti-GPⅠb monoclonal antibody h6B4-Fab (at a dose of 0.5 mg/kg) markedly reduced the reduction in blood flow in the stenosed femoral artery [74]. However, the success rate of drug development is low, and h6B4-Fab still needs to overcome potential challenges in clinical trials, such as dose optimization, long-term safety, and interactions with other drugs. Its production and purification may face technical difficulties, increasing the development cost.

*TaSER:* The TaSER fusion protein effectively disrupts platelet-driven thrombin production by binding to platelets and inhibiting clotting activity on their surface. Additionally, TaSER blocks vWF binding to platelets, inhibits platelet adhesion and aggregation, and limits thrombus reformation under flow conditions in vitro [75].

*AJW200:* AJW200 is an IgG4 monoclonal antibody directed against vWF. It inhibits platelet aggregation induced by high shear stress, the generation of thrombin and the adhesion of platelets to type III collagen [76]. Intravenous infusion of AJW200 dose-dependently inhibited vWF activity without prolonging skin bleeding time in a phase I clinical trial [77]. However, there are some limitations to AJW200. For example, its effect is limited under low-shear stress conditions, which may limit its application in some specific pathological states.

*82D6A3:* 82D6A3 is a monoclonal antibody targeting the vWF A3 domain. [78]. It inhibits platelet adhesion under shear stress, particularly under high-shear conditions in stenosed arteries, by blocking vWF binding to fibrillar collagen types I and III [79]. 82D6A3 has a high affinity for vWF (Kd = 0.4 nmol), and in baboons, 300mg of 82D6A3 completely prevented the reduction in blood flow in arterial constriction [78]. As a monoclonal antibody, 82D6A3 has a long half-life and rapid onset of action, making it suitable for the treatment of acute thrombotic disorders, such as ACS or stroke. Current research is mainly limited to animal models and early clinical trials, and there is a lack of large-scale clinical data to support its efficacy and safety.

*ARC1779:* ARC1779 is a nuclease-resistant aptamer which binds with high affinity to the vWF A1 domain and inhibits vWF-induced platelet aggregation. Clinical trial NCT00742612 showed that treatment with ARC-1779 rapidly reduced the frequency and mean intensity of TCD emboli [80]. However, ARC1779 has a short half-life (approximately 2 hours) and requires continuous infusion to maintain efficacy. Some clinical trials were suspended because of insufficient recruitment, which may have limited the completeness of their efficacy assessments.

*ARC15105:* ARC15105 is a second-generation aptamer that is an inhibitor of the vWF A1 domain. In a multicenter trial, ARC15105 (1.3µmol/L) completely inhibited ristocetin-induced platelet aggregation and partially inhibited collagen-, ADP-, arachidonic acid- and thrombin receptor-activated peptide-induced platelet aggregation [81]. Although ARC15105 has shown promising efficacy in laboratory studies, its safety still needs to be further verified.

***Rondoraptivon pegol (BT200)**:* BT200 is a novel pegylated aptamer derived from ARC15105 that targets the vWF A1 domain. In the blood of large artery atherosclerosis stroke (LAA) patients, BT200 reduced the mean vWF activity from 178 to less than 3% in a concentration dependent manner [82]. Although the primary mechanism of action of rondoraptivon pegol is to shorten the half-life of vWF by binding to its A1 domain, it is not known whether it affects vWF clearance in vivo through other pathways. Further studies may help to reveal its more comprehensive mechanism of action.

*TAGX-0004:* TAGX-0004 is a novel, highly-affine and specific DNA aptamer for the human vWF A1 domain and is at least 10 times more effective than ARC1779 in blocking vWF activity [83]. Preliminary studies have shown that TAGX-0004 did not lead to serious adverse events, including bleeding risk. However, as its mechanism of action is similar to existing therapies such as caplacizumab, further studies are needed to investigate its long-term safety and potential side effects.

*ALX-0081:* ALX-0081 has been designed to bind to the A1 structural domain of vWF. Studies in cynomolgus monkeys demonstrated that an intravenous bolus of ALX-0081 prevented the formation of thrombi induced by ristocetin and prevented femoral artery thrombosis with less increase in bleeding time compared to clopidogrel [84]. Despite the excellent performance of ALX-0081 in treating aTTP, its high research and development costs and manufacturing expenses may lead to high drug prices, thereby limiting its popularity and accessibility in developing countries.

*Caplacizumab:* Caplacizumab was evaluated in a clinical trial involving patients with TTP (NCT02553317), where it demonstrated lower rates of TTP-related death, recurrence, and thromboembolic events [85]. Despite the excellent performance of caplacizumab in terms of efficacy, its safety remains controversial. Common side effects of caplacizumab include mild bleeding events (e.g., epistaxis, skin ecchymosis), and severe side effects include fatal bleeding. Future studies should further verify its applicability and economy in different patient populations to optimize its clinical application strategy.

**Targeting CLEC-2**: Platelet CLEC-2 plays an essential role in the GPⅠbα-vWF-mediated signaling pathway leading to the activation of integrin αⅡbβ3 [86]. The absence of platelet CLEC-2 reduced the severity of both thrombosis and pulmonary thrombocytopenia in aTTP mouse model [86].

*2-Chlorophenol (2CP):* 2CP binds directly to CLEC-2 and specifically blocks PDPN/CLEC-2 and TCIPA interactions. It does not affect platelet aggregation induced by other platelet agonists [87]. However, due to its toxicity and the uncertainty of clinical translation, it is not yet possible to consider it as an effective clinical treatment. Future research should focus on clarifying its mechanism of action, optimizing its pharmacological properties, and verifying its safety and efficacy through rigorous clinical trials.

**Targeting 5-Hydroxytryptamine/Receptor Interactions**: 5-Hydroxytryptamine (5-HT), primarily found in platelet dense granules, acts as a weak platelet activator and enhances platelet aggregation elicited by various agonists [88].

*Sarpogrelate (MCI-9042):* A selective 5-HT(2A) antagonist, it reduces 5-HT and epinephrine-induced platelet aggregation in stroke patients in a dose-dependent manner [89]. As a new antiplatelet drug, sarpogrelate has shown significant clinical potential in improving PAD, diabetes-related diseases and heart diseases. However, its efficacy in fields such as ischemic stroke needs to be further verified. Future research should focus on improving the quality of research and exploring its application in more diseases.

*Temanogrel (APD791):* APD791 is an affinity selective 5-HT(2A) receptor antagonist [90]. It has been reported that in a dog model of coronary artery thrombosis, APD791 has reduced thrombosis without an increase in bleeding risk. If temanogrel successfully completes subsequent phase III trials and is approved, it may become a novel drug for the treatment of microvascular and cardiovascular diseases. However, its eventual market entry will depend on clinical trial results, regulatory approval, and competition with other existing therapies.

*5HT2ARAb:* 5HT2ARAb, an antibody that targets the 5-HT(2A) receptor, inhibits 5-HT-induced platelet activation in vitro. 5HT2ARAb prolongs the occlusion time of thrombi without affecting the bleeding time in the mice tail [91]. Despite the excellent antithrombotic performance of 5HT2ARAb, its safety and selectivity still need to be further evaluated. For example, it has been pointed out that 5HT2ARAb may exert effects on non-target cells, such as endothelial cells, which could lead to adverse effects. Moreover, because 5-HT2A receptor plays a role in a variety of physiological and pathological processes, targeting this receptor may trigger other side effects, such as psychiatric disorders or adverse cardiovascular effects.

**Targeting ACKR3**: Platelets express atypical chemokine receptor 3 (ACKR3), which acts to counteract platelet activation [36, 92]. Binding of macrophage migration inhibitory factor (MIF) and other ligands to ACKR3 can reduce thrombosis [92].

**Targeting adenosine A2A and A2B receptors**: Adenosine G-protein-coupled receptors (ARs) are located in the blood vessel wall and platelets. Through coupling with stimulatory G-proteins and adenylate cyclase (AC), activation of platelet ARs increases intracellular cyclic adenosine monophosphate (cAMP) levels, thereby inhibiting platelet activation and aggregation [93]. Some AR agonists, such as NECA, have demonstrated antiplatelet aggregation effects in vitro and antithrombotic activity in vivo [94].

**Targeting αIIbβ3 integrin**: Inhibition of the binding of fibrinogen to the activated αIIbβ3 integrin offers a platelet-specific approach to antithrombotic therapy [95, 96]. Recently developed αIIbβ3 integrin inhibitors can selectively inhibit the activation of αIIbβ3 integrin while not affecting nonactivated platelets, and in combination with conventional antithrombotic drugs can enhance the inhibitory effect on activated platelets while preserving the hemostatic function of non-activated platelets [97].

### 2.1.2 Antiplatelet drugs targeting intracellular signaling

Targeting PI3Kβ: PI3K is one of the signaling pathways that promotes shear-dependent platelet activation. Among these, PI3Kβ plays a particularly critical part in platelet functional responses elicited by ADP-P2Y12 interaction, GPⅥ conjugation, and αⅡbβ3 outside-in signaling.

*TGX-221:* Targeted blockade of PI3Kβ by TGX-221 significantly reduced FeCl_3_-induced carotid thrombosis in wild-type mice, without notably impairing hemostatic function [98, 99]. However, its clinical application still faces problems such as poor solubility, drug resistance and safety. TGX-221 is expected to become a more effective therapeutic tool by optimizing the delivery system, exploring the combination strategy and further studying its mechanism of action.

*AZD6482:* AZD6482 is a specific PI3Kβ inhibitor that prevents the interaction of PI3Kβ with ATP, thereby inhibiting platelet aggregation. In the modified Folt canine model, AZD6482 effectively prevented thrombosis without significant bleeding side effects. Its safety and efficacy were also verified in healthy male subjects [100]. Despite this, limited progress has been made in clinical trials of AZD6482. So far, only phase I clinical trials have been completed, and its safety and efficacy as an adjuvant treatment for ACS or stroke have not been fully verified.

*MIPS-9922:* MIPS-9922 is an effective inhibitor of PI3K-mediated platelet GP αⅡbβ3 activation and platelet adherence to vWF in vitro. In an electrolytic injury mouse model of AT, MIPS-9922 inhibited thrombosis without increasing bleeding time or blood loss [101]. Although the combination of MIPS-9922 and aspirin reduces the risk of bleeding, how to optimize the dose and medication regimen still need to be further explored.

**Targeting Syk**: Through GPⅥ and other signalling pathways, spleen tyrosine kinase (Syk) plays a critical part in platelet activation.

*Fostamatinib (R406):* Fostamatinib's active metabolite, R406, is a Syk inhibitor. R406 provides additional antiplatelet effects in patients already receiving aspirin or ticagrelor [102]. The safety of long-term fostamatinib needs to be further studied. Although no serious cumulative toxicity or thrombotic events were found in the current study, the potential risk to patients with cardiovascular disease remains a concern.

*BI1002494:* Treatment with BI1002494, a new, specific, and orally bioavailable Syk inhibitor, prevented AT in mice and resulted in smaller infarct sizes, leading to improved neurological outcomes 24 hours after transient MCA occlusion [103]. However, Van et al. showed that BI1002494 failed to completely inhibit GPVI-mediated thrombosis in humans [104]. This suggests that the use of BI1002494 in humans may require further dose optimization or combination with other antithrombotic agents to enhance efficacy.

**Targeting 12-LOX**: 12-lipoxygenase (12-LOX) is an enzyme primarily represented in human platelets and acts as a regulator of platelet activation [105]. Within platelets, 12-LOX is required for the activation of αⅡbβ3 as well as in platelet activation mediated by PAR4 and GPⅥ [106].

*ML355:* ML355 is a powerful, durable and selective inhibitor of 12-LOX that hinders thrombin- or TxA2-mediated platelet activation [107, 108]. It also inhibits platelet adhesion and thrombosis under human whole blood flow conditions [109]. Although ML355 did not significantly affect normal coagulation, it may trigger an inflammatory response in some cases. Further optimization of drug-delivery systems is needed to increase efficacy and reduce side effects.

**Targeting Bruton’s tyrosine kinase (BTK)**: BTK plays a role downstream of GPⅥ in collagen-induced platelet activation. BTK inhibition does not affect αⅡbβ1 integrin-mediated or vWF-mediated adhesion to collagen and thus normal hemostasis [110].

*AB-95-LH34:* AB-95-LH34 is a potent and highly specific BTK inhibitor that effectively blocks atherosclerotic plaque-induced thrombosis and GPⅥ-mediated platelet procoagulant activity in vitro. However, although it has shown highly effective antithrombotic activity in vitro, its efficacy and safety in humans still need to be verified by large-scale clinical trials.

*Acalabrutinib:* Acalabrutinib, a novel BTK inhibitor, inhibits the GPⅥ-dependent aggregation of blood platelets exposed to human plaque and collagen homogenates [110]. However, acalabrutinib may have lower cost-effectiveness than other BTK inhibitors.

*Evobrutinib:* Like acalabrutinib, it is a new generation BTK inhibitor with improved selectivity for BTK. Without adding to the risk of bleeding incidents, it was shown to inhibit GPⅥ-induced platelet activation and thrombosis [111]. Liver injury is one of the major safety concerns for evobrutinib. Despite its high incidence, most patients were able to continue treatment after dose modification. This issue has limited evobrutinib's widespread use and triggered the FDA's suspension of its clinical trials.

*Ibrutinib:* Ibrutinib, an irreversible BTK inhibitor, decreases platelet binding and thrombosis on human atherosclerotic plaques [112]. Additionally, ibrutinib reduced the occurrence of atherosclerosis in the middle cerebral artery in a high-fat diet-induced atherosclerosis model in rhesus monkeys [113]. However, its high price, drug resistance, and limitations in some indications still need to be further addressed. Future research should focus on optimizing treatment regimens, reducing economic burden, and developing new combination therapies to maximize patient benefit.

*Remibrutinib:* Remibrutinib (0.1 μM) markedly suppressed platelet aggregation caused by GPⅥ, vWF/GPⅠb and FcγRⅡA activation in vitro. It has demonstrated superior efficacy in inhibiting BTK-dependent platelet activation and hemostatic disorders [114]. Remibrutinib is more selective and less toxic than other BTK inhibitors such as ibrutinib. As a covalent irreversible BTK inhibitor, it can rapidly bind and permanently inactivate the BTK protein, thereby avoiding potential toxicity caused by long-term exposure. However, its long-term efficacy, indication expansion and competition with other therapies are still the focus of future research.

**Targeting PDI**: In mice, platelet PDI controlled clot size without affecting platelet adhesion or the production of fibrin at sites of arterial injury. Platelet-selective PDI knockout mice had impaired thrombogenesis and recombinant PDI infusion restored cremaster muscle thrombosis without significantly prolonging tail bleeding time [115].

*RL90:* ERp57, a closely related homologue of PDI, is also found on the platelet cell surface. RL90, an antibody targeting ERp57, strongly inhibits ERp57 activity and platelet aggregation. RL90 effectively inhibited FeCl_3_-induced thrombosis but prolonged tail bleeding time [116]. However, the mechanism of action of RL90 may be influenced by other factors. For example, it has been pointed out that the inhibitory effect of RL90 on platelet activation may be related to the inhibition of protein disulfide isomerase. In addition, RL90 may not completely inhibit platelet activation under certain experimental conditions, suggesting that it may have limitations in clinical use.

*Quercetin-3-rutinoside (K3R):* K3R is a selective inhibitor of PDI. It did not increase bleeding time and blocked FeCl_3_-induced AT in mice [117]. In addition, K3R was also shown to reverse thrombosis caused by chlorotoxins, further validating its antithrombotic potential [118].

*Isoquercetin:* Isoquercetin reduced PDI-mediated FⅤ activation by 75% [119]. A phase Ⅱ study (NCT02 195232) showed that oral isoquercetin reduced the levels of plasma D-dimer, platelet-derived thrombin generation and circulation P-selectin in cancer patients at high thrombosis risk [120]. However, since its mechanism of action is not completely clear and some research results are contradictory, further research is still needed to fully explore its clinical value. Future research should focus on mechanism analysis, production process optimization and large-scale clinical validation to promote the wide application of isoquercetin in the medical field.

*Myricetin:* Myricetin is a potent inhibitor of collagen and TRAP-6-induced platelet aggregation, adhesion to collagen, thrombi formation, and adhesion of fibrinogen to human platelets under flow conditions [121]. The poor water solubility and low bioavailability of myricetin limit its clinical effectiveness. At present, scientists are improving its solubility and absorption through chemical modification and nanotechnology.

**Targeting PDK1**: PDK1 regulates αⅡbβ_3_ integrin-dependent outside-in signaling by inhibiting glycogen synthase kinase 3β through an AKT-dependent pathway, which influences thrombin-induced platelet clot aggregation.

*BX795:* BX795 blocked 2-mesADP-and collagen-induced platelet aggregation, ATP and thromboxane production by inhibiting PDK1, ultimately leading to reduced clot retraction [122]. Although BX795 shows high selectivity for PDK1 with an IC50 value of 6 nM, it also significantly inhibits other kinases, such as TBK1 and IKKε. BX795 acts through a variety of signaling pathways, and its complex regulatory mechanism may increase the difficulty of clinical application [123].

**Targeting CK2**: Casein kinase 2 (CK2) is a constitutively activated tetrameric serine/threonine protein kinase that interacts closely with the PI3K-AKT signaling pathway [124, 125]. CK2β^-/-^ mice exhibited significantly reduced thrombosis and stabilization at high arterial shear rate and attenuated thrombotic vascular thrombosis. After MCAO, CK2β^-/-^ mice had significantly smaller infarction volume and considerably reduced neurological deficits [126].

*CX-4945:* CX-4945 inhibition effectively blocked ADP-and PAR1 peptide-induced platelet aggregation in vitro by inhibiting CK2 activity. Furthermore, its antithrombotic effect has been confirmed in a model of thrombosis induced by a photochemical process in mice [127]. However, its complex mechanism of action and multi-target characteristics may bring potential therapeutic risks. For example, its effects on other kinases or signaling pathways other than CK2 may trigger unpredictable side effects.

*TBCA:* TBCA is a highly selective CK2 inhibitor. In the presence of TBCA, the spreading of platelets on immobilized fibrinogen and the retraction of the clot, both mediated by integrin αⅡbβ_3_ signaling, were markedly inhibited [128].

**Targeting SGK1**: SGK1 is a serine/threonine protein kinase downstream of PI3K-PDK1 signaling. Upregulation of Ca²^+^ release channels by SGK1 in platelets is associated with thrombosis [129].

*GSK-650394:* GSK-650394 is an SGK1 inhibitor that may have a neuroprotective effect. Inhibition of SGK1 with GSK-650394 during the time window of thrombolytic therapy attenuates cerebral I/R injury [130]. However, long-term use may lead to exacerbation of inflammatory reactions or other adverse effects [131].

Targeting Annexin A7: There was no increase in bleeding when *Anxa7* was deleted in mice. Instead, it significantly reduced collagen-induced platelet aggregation and provided a protective effect against AT [132].

*ABO:* ABO is a small molecule targeting annexin A7.ABO-treated mice showed protection against collagen-induced thrombosis without affecting hemostasis, compared to control mice [132].

### 2.1.3 Antiplatelet drugs targeting metabolism

The transition of platelets from a quiescence to an activated state is characterized by a significant increase in glycolysis [133, 134]. Since platelet activation is a high-energy process that accelerates ATP production through aerobic glycolysis, aerobic glycolysis is faster than OXPHOS [135, 136]. Thus, targeting metabolites or key metabolic enzymes can have a major impact on platelet activation and clotting [133, 134, 137].

**Targeting pyruvate dehydrogenase kinases (PDK)**: During platelet activation, PDK phosphorylates and inhibits PDH E1α subunit activity. This inhibition redirects the flow of pyruvate from the Krebs cycle to aerobic glycolysis [134, 138].

*DCA:* At arterial shear rates, DCA-treated human blood perfused ex vivo on collagen formed markedly smaller thrombi in a microfluidic flow chamber [138]. DCA treatment also attenuated platelet function stimulated by multiple agonists and greatly slowed the time to closure of mesenteric arterioles in mice after FeCl_3_-induced injury [133].

**Targeting PKM2**: Pyruvate kinase (PK) exists in mammals in four isoforms-PKL, PKR, PKM1, and PKM2-each with tissue-specific expression. The dimeric form of PKM2 has low enzymatic activity, allowing it to act as a metabolic switch that regulates aerobic glycolysis. Activation of human platelets is associated with an increase in the expression of dimeric PKM2, which is supportive of this metabolic shift [137].

*ML265:* ML265 is a low molecular weight inhibitor that prevents PKM2 dimer formation and reduces aerobic glycolysis [139]. In a mouse model of carotid and mesenteric artery thrombosis, platelet-specific PKM2 knockout decreased platelet activation and thrombosis [137]. ML265 may act by affecting multiple metabolic pathways, such as glycolysis and amino acid synthesis, which adds to the complexity of its mechanistic studies [140]. Future research can focus on optimizing its formulation, exploring new delivery systems, and understanding its mechanism of action to promote its clinical application.

*DASA:* In vitro thrombin (0.5 U/mL)-induced platelet aggregation was reduced by 58% by treatment with DASA (200 mM), a small molecule modulator of PKM2. However, extravasation of red blood cells was observed in the PE mouse model after DASA treatment, and further evaluation of tail hemorrhage also showed prolonged bleeding time and increased bleeding volume [133]. However, its practical application still faces challenges such as toxicity and drug resistance. Future studies should focus on optimizing its structural design, clarifying their mechanism of action, and evaluating their long-term safety to promote their widespread use in clinical medicine.

**Targeting β-Oxidation of fatty acids**: In the energy metabolism of platelets, fatty acids play a key role [141]. β-oxidation of fatty acids in mitochondria can produce ATP via OXPHOS [142]. This process has been shown to contribute to thrombin-induced ATP production in platelets [143].

*Etomoxir:* Etomoxir reduces platelet oxygen consumption rate by inhibiting endogenous fatty acid β-oxidation and increases extracellular acidification rate (ECAR), suggesting that platelets depend on fatty acids for ATP production [143]. Although etomoxir has shown potential in multiple areas, its toxicity issues have limited its widespread use. Etomoxir raised serious hepatotoxicity concerns in patients with heart failure and led to early termination of clinical trials [144].

**Targeting Glutamine oxidation**: Glutaminolysis is the process in which glutamine is converted into Krebs cycle metabolites, through the action of transglutaminase. This process supports mitochondrial OXPHOS [145].

*Azaserine:* Treating thrombocytes with azaserine, a glutamine structural analogue, decreased OCR, indicating that glutamine plays a role in supporting mitochondrion respiration [143]. Despite the multiple biological effects of azaserine, its toxicity cannot be ignored. In animal studies, azaserine can cause DNA damage and carcinogenesis in the pancreas, liver and kidney. In addition, the rapid metabolism of azaserine in humans may lead to limited therapeutic efficacy. In clinical applications, it is necessary to strictly control the dose to avoid serious toxic and side effects.

The mechanism of action of antiplatelet drugs mainly includes the inhibition of platelet surface receptors (such as GPVI, PARs, ADP receptors) and intracellular signaling pathways (such as PI3K). Inhibitors of these novel drug targets can reduce thrombosis while having less impact on normal hemostasis, which provides a new direction for the development of safer and more effective antiplatelet drugs. However, these inhibitors are still in the early stage of development, and future studies need to further optimize drug dosage and regimen to reduce adverse reactions. The development of new antiplatelet drugs with more selective and less toxic side effects is an important goal in the future.

**Figure 5.**
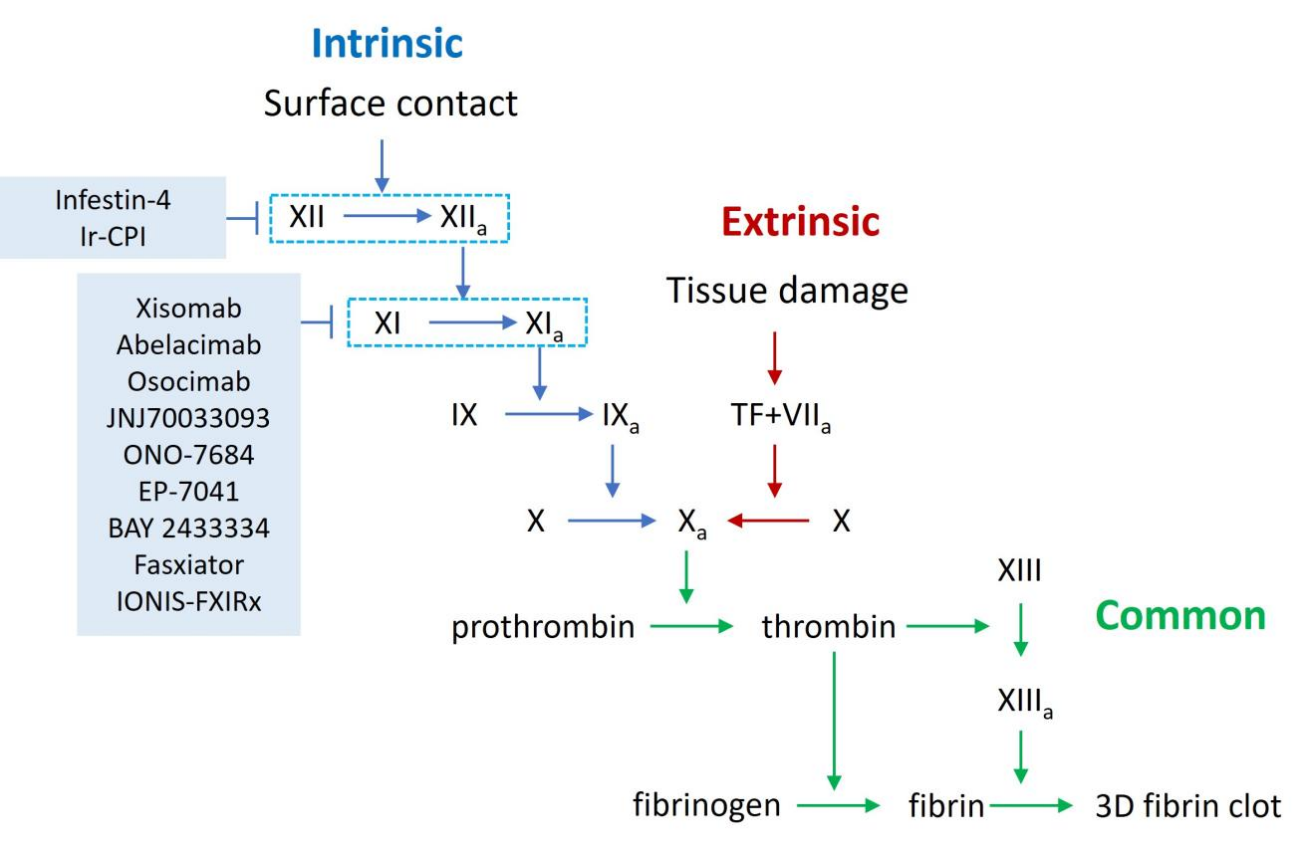
**Coagulation cascade and new targets for anticoagulant therapy**. The coagulation pathway is divided into the intrinsic (endogenous) and extrinsic (exogenous) pathways. Deficiency of FⅫ and FⅪ, which are key molecules in the contact system of the intrinsic pathway, does not result in bleeding disorders. This has made them attractive new targets for anticoagulant therapy, as inhibiting these factors could prevent thrombosis without increasing the risk of bleeding.

### 2.2 New targets for anticoagulant therapy

The coagulation cascade is a process in which a series of coagulation factors are activated one after another, which plays a central role in thrombosis. It is mainly achieved by initiation, amplification, formation of thrombus framework and stabilization of thrombus. There are two ways to initiate the coagulation process [146]. One is the endogenous coagulation pathway. When the vascular endothelium is damaged and the subendothelial collagen fibers are exposed, FXⅡ in the blood comes into contact with them and is activated, thus initiating the endogenous coagulation pathway. After FXⅡa activation and FXⅠa were activated in turn, which laid the foundation for the subsequent coagulation reaction. The other is the initiation of an exogenous coagulation pathway. During vascular injury, TF is exposed and binds to FⅦ in the blood to form TF-FⅦ complex, which rapidly activates FX and initiates exogenous coagulation pathway. The exogenous coagulation pathway is rapid and can initiate the coagulation process in a short time after injury, which plays an important role in the process of wound hemostasis and AT [147]. The second step is to amplify the coagulation reaction. Both endogenous and exogenous coagulation pathways ultimately converge on the activation into FX, which, upon activation to FXa, forms a prothrombin complex with FVa, calcium ions, and platelet phospholipids. The prothrombin complex converts prothrombin into active thrombin. In addition to converting fibrinogen into fibrin monomer and promoting the formation of thrombus framework, thrombin can also feedback activate coagulation factors Ⅴ, Ⅷ, Ⅺ, etc. to further amplify the coagulation reaction, so that more coagulation factors participate in the coagulation process and accelerate the formation of thrombosis. The third step is the formation of the thrombus framework. Thrombin converts fibrinogen to fibrin monomers, which, in response to FⅩⅢa, rapidly cross-link to form fibrin multimers. Fibrin multimers interweave with each other into a network structure, which integrates various blood cell networks such as red blood cells, white blood cells, and platelets in the blood to form the basic framework of thrombosis. This is the key step of thrombosis, and the thrombus begins to have a certain morphology and structure. The last step is to stabilize the thrombus structure. Thrombin activates FXⅢ to FXⅢa, FXⅢa can make more covalent bonds between fibrin monomers, strengthen the cross-linking between fibrin polymers, make the fibrin network more stable, enhance the strength and stability of the thrombus, and prevent the thrombus from being dispersed by blood flow during the formation process [148]. Thrombin can activate platelets and promote platelet aggregation and release reactions. Platelet aggregates are further filled in the fibrin network, which increases the density of the thrombus. At the same time, a variety of substances released by platelets can further promote the coagulation cascade, so that the thrombus continues to grow and stabilize.

Targeting contact systems for anticoagulation can yield agents with fewer bleeding complications and low renal clearance. Contact coagulation factors include classical FⅫ, FⅪ and HMWK of the kinin system. Their common feature is initiating the endogenous coagulation pathway via contact reactions and being linked to systems like kinin, fibrinolysis, and complement. Targeting FXII or FXI to reduce side effects is the focus of current research. Therefore, drugs or preparations are summarised in Figure 5 and Supplementary Table 4. For example, targeting FXII or FXI has been shown to limit thromboembolic complications associated with ECMO [149].

### 2.2.1 Targeting FXIIa

FXIIa is a coagulation factor. It plays a primary role in the first step of contact activation and is activated when blood contacts a negatively charged surface. Activated FXIIa can initiate a series of coagulation reactions and eventually facilitate the formation of fibrin clots which is of great significance to the coagulation process. However, FXIIa deficiency does not typically cause severe bleeding symptoms.

*Infestin-4:* rHA-infestin-4 is an inhibitor of coagulation FXIIa. In both mouse and rabbit models of mechanically-induced arterial and FeCl_3_-induced VT, rHA-infestin-4 could prevent occlusion and protect against thrombosis [150]. Since Infestin is derived from insect proteins, its immunogenicity in humans may become an important factor limiting its clinical application. Although its half-life was prolonged by fusion with human albumin, whether this strategy is sufficient to overcome the immunogenicity problem requires further investigation.

*Ir-CPI:* Recombinant Ir-CPI a contact-phase inhibitor, can specifically interact with FXIIa, FXIa and kallikrein. Intravenous administration of Ir-CPI dose-dependently inhibited VT in rats and mice and also prevented thromboembolism induced by collagen and adrenaline without promoting bleeding [151]. Currently, Ir-CPI is still in the early stage of clinical trials (such as phase Ⅰ and Ⅱa), and the sample size is small. More large-scale and multi-center clinical trials are needed to verify its long-term efficacy and safety.

### 2.2.2 Targeting FXIa

FXIa is a special target for antithrombotic drugs because its role in thrombosis is more critical than its role in hemostasis. This leads to the conclusion that inhibition of FXIa will inhibit thrombosis without interfering with normal hemostasis. FXIa is involved in a positive feedback mechanism in thrombosis. In addition to its ability to convert fibrinogen to fibrin, thrombin can feedback-active FXIa during thrombosis [152]. The activation of FXIa further accelerates the production of thrombin, and this positive feedback mechanism makes the coagulation process continuously amplified, thereby promoting the formation and development of thrombosis. However, in contrast to the thrombosis process, in the normal hemostasis process, this positive feedback is limited by a variety of regulatory mechanisms of the body and will not be overactivated. FXIa can also interact with platelets to promote thrombosis [153]. FXIa can bind to receptors on the surface of platelets and promote platelet activation and aggregation. Platelet aggregation plays a central role in thrombosis and forms platelet thrombi, which provides the basis for subsequent fibrin deposition and thrombus stabilization. While platelets are involved in both processes, the interaction between FXIa and platelets is more significant and critical during thrombus formation [154]. In addition, under some pathological conditions, such as atherosclerosis and inflammation, the damage of vascular endothelial cells and the exposure of subendothelial components can lead to the abnormal activation of FXIa, which then initiates the coagulation cascade and promotes thrombosis. Conversely, during normal hemostasis, vascular injury is relatively limited, and the activation of FXIa is within a controllable range.

FXIa can exert thrombotic effects in the absence of TF, mainly through the activation of the endogenous coagulation pathway, and this process is closely related to its dimer structure. FXIa is a plasma glycoprotein that exists mainly as a dimer. The monomer is a single-chain polypeptide composed of 607 amino acids with a relative molecular mass of about 80kDa [155]. The two monomers are linked by disulfide bonds to form a dimer structure, which is essential for the proper function of FXIa. The dimeric structure of FXIa helps to maintain its stability and activity in the blood circulation. During activation of the endogenous coagulation pathway without the involvement of TF, FXIa needs to remain intact to be correctly recognized and bound by activators such as FXIIa or kallikrein [156]. The interaction between amino acid residues within the dimer structure, such as hydrogen bonding and hydrophobic interaction, reduces the flexibility of the molecule, reduces the possibility of degradation or denaturation in the physiological environment, and ensures that FXIa can participate in the thrombosis process in the active form. As a serine protease, the dimer structure of FXIa can regulate the enzyme activity by affecting the conformation of the active center. In the dimeric state, the interaction between the two subunits is able to fine-tune the position and orientation of amino acid residues in the active center to a state suitable for substrate binding and catalytic reactions. When FXIa is activated by an activator, changes in the dimer structure will further expose the active center and enhance its affinity for substrates, such as FIXa, thereby facilitating the coagulation reaction and driving thrombosis. The dimeric structure of FXIa provides multiple interaction interfaces that allow it to interact with a variety of proteins, playing a key role in thrombosis without the involvement of TF. For example, FXIa can bind to high molecular weight kininogen, and this binding may affect FXIa activation. In addition, FXIa may also bind to receptors on the surface of platelets through its dimer structure to promote platelet activation and aggregation, which is an important link in thrombosis.

*Xisomab (AB023):* Xisomab is an antibody specifically used to inhibit the activation mediated by FXI. A phase II study (NCT03612856) showed that the using AB023 to inhibit coagulation induced by contact activation was well tolerated in patients with end-stage renal disease undergoing haemodialysis [157]. Early studies showed that it provided durable anticoagulation and had a favorable safety profile. However, data on the long-term use of xisomab are lacking. For example, its potential side effects such as immunogenicity and drug resistance in long-term use still need to be further studied.

*Abelacimab (MAA868):* Abelacimab is a high-affinity antibody targeting FⅪ. Abelacimab binds to FⅪ, inhibiting its activation. Abelacimab showed good anticoagulant activity in both mouse and cynomolgus monkey models of thrombosis, and subcutaneous abelacimab was clinically well tolerated [158]. Abelacimab may increase the risk of ischemic stroke in some studies, such as the increased rate of ischemic stroke observed in OCEANIC AF [159].

*Osocimab (BAY1213790):* Osocimab works by binding to the catalytic structural domain of FⅪa and blocking its activity. In phase II study (NCT03276143), osocimab achieved similar therapeutic effects to enoxaparin at a minimum of 0.6 mg/kg [160]. However, osocimab did not significantly reduce VTE compared with apixaban at some doses, with similar bleeding risks [161].

*JNJ70033093 (BMS-986177):* JNJ70033093 inhibits FⅪa activity by reversibly binding to its active site. In the post-operative period following knee replacement surgery, oral JNJ70033093 was effective in preventing VTE with a lower risk of bleeding compared to enoxaparin (NCT03891524) [162]. At present, there is no antidote for JNJ-70033093 overdose, and measures such as activated carbon should be taken to reduce absorption in the case of overdose. Further studies are needed to fully evaluate its long-term efficacy and safety, and to address potential risks.

*ONO-7684:* ONO-7684 is an inhibitor of activated FⅪa. A clinical trial in healthy subjects showed that the drug was well tolerated with a low incidence of adverse effects and no increased risk of bleeding [163]. Although ONO-7684 showed promising safety and efficacy in early studies, further studies are needed to verify its efficacy and safety in specific patient populations, such as patients with cardiovascular disease or cancer. In addition, its bleeding time prolongation effect compared with other FXIa inhibitors in some animal models still needs to be further evaluated [164].

*EP-7041:* Ideal for use in critical care, EP-7041 is a small molecule inhibitor of FⅪa [165]. However, despite the excellent performance of EP-7041 in animal models, its long-term efficacy and safety in humans need to be further observed. For example, some studies point to potential risks associated with the rapid introduction of new anticoagulants into clinical practice without adequate validation [166].

*BAY2433334:* Similar to JNJ70033093, BAY2433334 is an FⅪa inhibitor that targets its active site. The PACIFIC-STROKE trial (NCT04304508) compared the efficacy of BAY2433334 taken once daily with placebo. The results showed that BAY2433334 did not reduce mortality [167]. Although BAY2433334 has shown potential in some indications, its development process has faced challenges. For example, in the OCEANIC-AF phase III study, the drug failed to meet expectations due to poor efficacy. In addition, the market competitiveness of BAY2433334 may be limited compared with existing anticoagulants, such as rivaroxaban and apixaban.

*Fasxiator:* Fasxiator is a FXIa specific inhibitor. In vitro experiments showed that the engineered rFasxiatorN17R was 1000 times more potent, prolonged carotid artery occlusion in mice and had enhanced anticoagulant activity in human plasma [168]. FasxiatorN17R and L19E were formed by modification on the basis of fasxiator, with improved FXIa affinity (Ki = 0.9 nM) and selectivity. Specific attenuation of the coagulation pathway in vivo persisted for at least 60 minutes after intravenous administration of rFasxiatorN17R, L19E in rats. In AT model, intravenous administration of 2 mg/kg rFasxiatorN17R and L19E can achieve antithrombotic effect similar to that of unfractionated heparin (UFH), and can shorten the bleeding time by more than 3 times [169]. As a novel anticoagulant candidate, Fasxiator and its derivatives have shown promising antithrombotic effects and low bleeding risk in animal models. However, its application in humans still needs to overcome technical obstacles such as low production efficiency and poor stability. Future research should focus on optimizing the manufacturing process, improving the stability of the drug, and conducting human trials to verify its clinical value.

*IONIS-FXIRx:* IONIS-FXIRx is an antisense oligonucleotide (ASO) that targets FⅪ. In a phase II study (NCT02553889) involving 43 patients with ESRD undergoing haemodialysis, IONIS-FXIRx lowered patients' mean FⅪ activity by more than half compared to placebo. Despite all patients receiving heparin, patients treated with IONIS-FXIRx experienced fewer bleeds [170]. Because FXI inhibitors require long-term administration, the gradual recovery of FXI activity after drug discontinuation may limit their use in certain acute or short-term treatment scenarios.

*Pentagalylglucoside sulfated:* An allosteric inhibitor of FXIa, pentagalylglucoside sulfated, is a sulfated aromatic mimetic of heparin with a specificity of 551 nM for FXIa, which is at least 200-fold more selective than other related enzymes [171]. The current research mainly focuses on laboratory and animal models, and there is no large-scale clinical trial data to support its application in human patients. Future research should focus on optimizing its clinical application strategy and verifying its efficacy and safety through large-scale clinical trials.

*Sulfated chiral inositol (SCI):* Allosterism caused by heparin-binding sites on thrombin is a promising approach to produce safer drugs. SCI, a non-glycoheparin mimetic, inhibited FXIa by 280 nmol/L, which could be rapidly reversed by common toxic agents such as protamine. In rats, studies of FeCl_3_-induced AT and thromboplastin induced VT models showed that SCI at 250 μg/rat reduced thrombosis, matched by enoxaparin at 2500 μg/rat [172]. SCI, as a chiral compound, has great clinical significance in drug development and disease treatment. Its unique chirality and sulfation modification allow it to exhibit unique advantages in pharmacological activity, metabolic properties, and safety. However, the current research still has limitations, and its application potential and safety in different diseases need to be further explored.

### 2.2.3 Targeting other coagulation factors

Allosteric inhibitors play an important role in antithrombosis by binding to the allosteric sites of key targets in thrombosis, such as coagulation factors, thereby inducing conformational changes in the target to regulate its activity. Currently, some small molecules, peptides, biological macromolecules, and nanoparticles are being developed as allosteric inhibitors to exert antithrombotic effects.

*Sulfated pentagaloyl glucopyranoside (SPGG):* FXa is a key enzyme in the coagulation cascade. Allosteric inhibitors can bind to the allosteric site of FXa, induce a conformational change in its active center, and make it unable to effectively bind to the substrate, thereby blocking the conversion of prothrombin to thrombin, inhibiting the coagulation process, and playing an antithrombotic effect. SPGG is a kind of sulfated non-glycosaminoglycan mimetic. It does not affect the activity of coagulation factors other than FXa. However, its anticoagulant potential is reduced by serum albumin and FXI, and this effect can be reversed by protamine or polybrene [173]. Despite these limitations, the sulfation degree of SPGG significantly affects its inhibitory ability against FXIa. It was found that the inhibitory effect may be attenuated when the proportion of unsulfated molecules is higher. Future efforts should focus on finding more efficient and stable inhibitors by synthesizing and screening SPGG derivatives with different sulfation modes.

*KB-FVIIa-004:* An anti-FVIIa scFv, KB-FVIIa-004, was screened from an alpaca phage library. In FVIII-deficient mice, KB-FVIIa-004 was validated to block FVIIa activity in the absence of TF. A dose of 3 mg/kg FVIIa effectively corrected bleeding in FVIII-deficient mice, but bleeding was increased in mice that received 3 mg/kg FVIIa in the presence of KB-FVIIa-004 [174]. However, aithough KB-FVIIa-004 can specifically bind to and inhibit FVIIa, whether it will have an effect on other FVIIa-dependent physiological processes needs to be further studied. For example, FVIIa is not only involved in blood coagulation, but may also play a role in immune regulation and inflammatory responses. Therefore, its potential effects on these non-coagulation functions need to be carefully evaluated in clinical application [166].

*DNA aptamers:* Allosteric inhibitors can bind to specific allosteric sites of thrombin, change the structure of its active center, prevent the cleavage of fibrinogen and the activation of coagulation factors, thereby inhibiting thrombosis. Based on this mechanism of allosteric inhibitors, by screening a single-stranded DNA (ssDNA) library of predefined DNA nanostructures, a group of super-potent bivalent aptamers targeting human α-thrombin were screened. These aptamers allosterically attenuated thrombin cleavage activity strongly and showed an extremely potent anticoagulant effect in human plasma, demonstrating their great potential for therapeutic applications [175].

*RNA aptamers:* RNA aptamers possess the ability to specifically target individual coagulation factors, endowing them with the potential to serve as both anticoagulants and probes for exploring structure-function relationships in the coagulation system. Specifically, by zeroing in on distinct coagulation factors, RNA aptamers can precisely modulate the coagulation cascade, which is crucial for developing effective anticoagulant therapies and understanding the intricate mechanisms of blood coagulation. Notably, an RNA aptamer has been reported to utilize exosome-dependent allosterism, a mechanism where exosomes (small extracellular vesicles) facilitate conformational changes in a target protein (in this case, FIXa) at a site other than the active site, to achieve specific inhibition of FIXa [176].

New anticoagulants, including direct thrombin inhibitors and FXa inhibitors (some of which are new oral anticoagulants, NOACs), have demonstrated higher efficacy and safety in clinical applications. However, since the molecules in the contact system play a more crucial role in thrombosis than in hemostasis, the development of their inhibitors is expected to better address the side-effect of bleeding, which has become a current research hotspot. For example, Abelacimab, as the first FXI inhibitor to substantially reduce major bleeding, has received FDA fast-track status, which highlights the potential of contact system inhibitors in improving the safety of anticoagulant therapy. Future research will focus more on achieving a balance between efficacy and safety, realizing individualized treatment, and applying emerging technologies.

### 2.3 New targets for anti-inflammatory therapy

**Figure 6.**
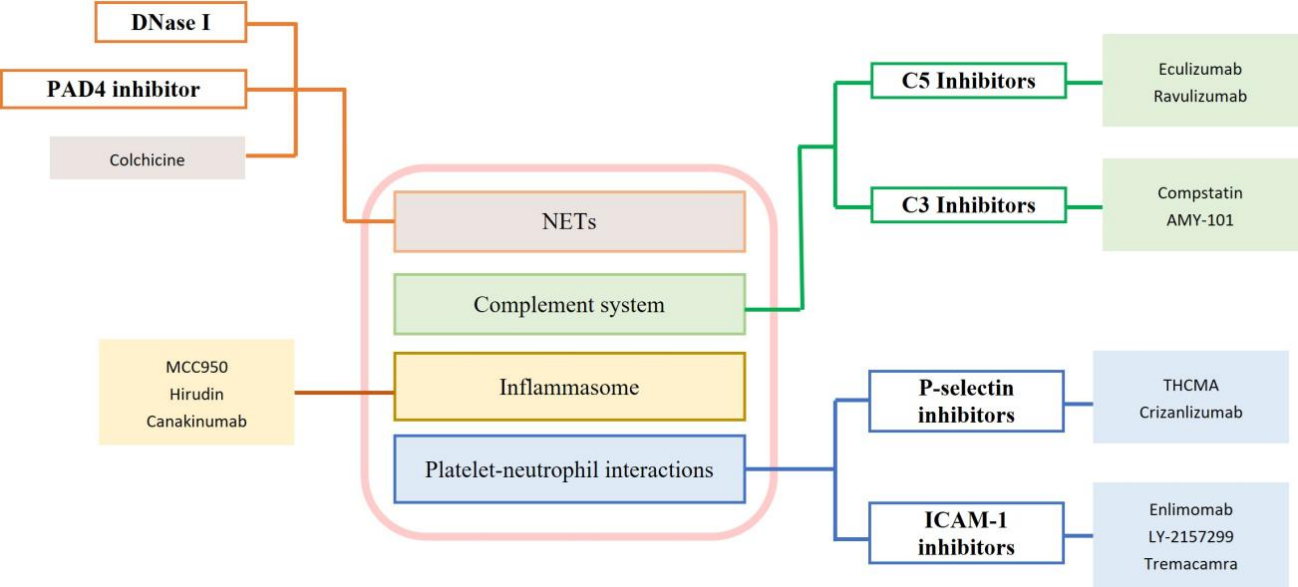
**New targets for anti-inflammatory therapy**. According to the type of target, it can be divided into NETs, complement system, inflammasome and platelet-neutrophil interactions.

In an inflammatory environment, vascular damage exposes the subendothelial matrix, releasing stimulatory factors that activate platelets. These platelets can quickly accumulate at the injury site and direct the formation of a blood clot. Proinflammatory cytokines amplify platelet hemostatic functions and accelerate the coagulation process. During inflammation-associated thrombosis, neutrophils form NETs, which act as scaffolds that promote thrombosis by retaining and activating platelets, thereby impeding blood flow and contributing to thrombus formation [177]. The complement system is one of the core mediators of the innate immune defence, which promotes thrombosis by activating platelets or coagulation pathways [178]. Inflammasomes further drive thrombosis progression by inducing endothelial cell injury and activating macrophages. In addition, platelet-neutrophil interaction plays a key role in the thrombosis process. These processes represent potential therapeutic targets for future thrombosis treatments, and the targets and agents that target inflammation are summarized in Figure 6.

### 2.3.1 Targeting NETs

Neutrophils can release chromatin filaments covered with nuclear proteins, known as NETs [179]. Neutrophil NETosis has emerged as a key mechanism in atherosclerotic inflammation.

*Deoxyribonuclease I (DNase I):* DNase I is an enzyme that breaks down the DNA and thus breaks down the NETs. The addition of DNase I to standard t-PA therapy improved thrombolysis in thrombi collected from ischemic stroke patients undergoing endovascular treatment [180]. In a I/R injury mouse model, chromatin removal via DNase I treatment provided cardio-protection [181].

*PAD4 Inhibitor:* A key enzyme in neutrophils responsible for the production of NETs is protein arginine deiminase 4 (PAD4). The effects of inhibiting PAD4 and NOX on cerebral microcirculation were evaluated in a mouse model of photoactivated thrombosis. The results suggest that the inhibition of PAD4 and NOX may impede cerebral thrombosis by reducing pathological histone H3-citrullinated neutrophils [182].

*Colchicine:* COLCOT trial shows colchicine significantly reduces risk of MI and stroke [183]. Colchicine's benefit lies in its ability to inhibit NET formation and attenuate NLRP3 inflammasome activation [184, 185]. In the LoDoCo2 study, colchicine significantly reduced inflammatory mediators within 30 days in patients with a history of MI [186]. However, despite the wide clinical utility of colchicine, its potential toxicity cannot be ignored. High doses of colchicine may cause gastrointestinal reactions such as diarrhea, nausea, vomiting, and even renal damage in severe cases. Given its complex mechanism of action and large individual differences, dose adjustment and medication monitoring of colchicine are particularly important to minimize the risk of toxicity [187].

### 2.3.2 Targeting complement system

Activating complement can promote thrombosis. On the one hand, it activates coagulation factors, on the other hand, it promotes platelet aggregation, and these effects together participate in the process of thrombosis.

**C5 Inhibitors**: C5 serves as a pivotal component within the complement system, playing a significant role in the amplification and execution of the complement-mediated immune response. Specifically, upon complement activation, C5 is cleaved into C5a and C5b. C5a is a potent anaphylatoxin that triggers inflammation by recruiting immune cells and promoting the release of inflammatory cytokines, while C5b initiates the formation of the membrane-attack complex (MAC), which can cause cell lysis and also contribute to a pro-thrombotic state. Numerous studies have demonstrated that C5 inhibitors can effectively reduce the risk of thrombotic diseases. These inhibitors work by blocking the cleavage of C5, thereby suppressing the generation of C5a and C5b. As a result, they mitigate both the inflammatory effects induced by C5a and the pro-thrombotic effects associated with MAC formation, ultimately lowering the likelihood of thrombotic events.

*Eculizumab (Soliris):* Eculizumab is an anti-C5 antibody used to treat diseases associated with abnormal complement system activation. Clinical trials have shown that eculizumab is strongly correlated with reduced mortality and recurrences of thrombotic events in vascular liver disease (VLD) and paroxysmal nocturnal haemoglobinuria (PNH) patients [188]. However, the high cost, risk of infection and safety of long-term use still need to be further studied and solved. Future studies should focus on indication expansion, individualized treatment regimens, and the development of combination therapies to maximize the clinical value of eculizumab and reduce its potential risks.

*Ravulizumab:* Ravulizumab, a long-acting derivative of eculizumab, is another anti-C5 antibody. With a longer half-life than eculizumab, ravulizumab allows for less frequent administration. It represents a new therapeutic target and treatment option for thrombotic microangiopathy [189]. The safety profile of ravulizumab is generally favorable, and adverse effects are mostly mild to moderate. However, since ravulizumab inhibits the complement system, which is an important part of the body's immune defense against pathogens, patients may be more susceptible to certain infections, such as those caused by Neisseria spp. Moreover, long-term use of ravulizumab may require regular monitoring of complement levels. This is because monitoring can help clinicians assess the drug's therapeutic efficacy in controlling the over-activation of the complement system and also identify potential risks, such as an increased risk of infection due to excessive complement inhibition.

**C3 Inhibitors**: Complement C3 plays a pivotal role in promoting thrombosis through well-defined mechanisms. Specifically, it can bind to specific receptors on platelets, triggering intracellular signaling pathways that lead to platelet activation and subsequent aggregation. Additionally, C3 fragments can interact with coagulation factors, enhancing their activation and accelerating the coagulation cascade. Given the significant role of C3 in thrombosis, it is reasonable to explore the potential of C3 inhibitors as therapeutic agents. Several studies have indicated that C3 inhibitors may work by blocking the cleavage of C3 or interfering with the binding of C3 fragments to their receptors. As a result, they can potentially disrupt the processes of platelet aggregation and coagulation factor activation, offering promising therapeutic benefits in preventing thrombosis and its associated complications.

*Compstatin:* Compstatin is a synthetic peptide with high selectivity for inhibiting C3 activation. For instance, treatment of neutrophils with COVID-19 platelet-rich plasma were found to induce TF-carrying NETs that promote thrombotic activity in human aortic endothelial cells (HAECs). Inhibiting complement C3 using compstatin Cp40 abolished neutrophil TF expression [190]. The early versions of compstatin limited its clinical use due to rapid renal excretion. This problem has been significantly ameliorated by the new generation of derivatives through structural optimization, such as the introduction of lysine or pegylation [191]. The broad role of the complement system may bring potential side effects, for example, in systemic diseases such as systemic inflammatory response syndrome (SIRS) and sepsis, excessive inhibition of complement may trigger new immune problems.

*AMY-101:* AMY-101 is a novel C3 inhibitor known for its highly selective inhibition of C3 activation. AMY-101 has successfully treated patients infected with SARS-CoV-2 and those with post-thrombotic ARDS [192]. However, several studies have pointed out that AMY-101 may activate the alternative pathway of the complement system in a non-convertase-dependent manner, which may affect its efficacy in some cases. Therefore, its impact on the alternative pathway needs to be carefully evaluated in practical applications [193].

### 2.3.3 Targeting inflammasome

The activation of inflammasomes sets off a complex cascade of events that ultimately promotes thrombosis. Specifically, upon activation, inflammasomes induce an inflammatory response by facilitating the cleavage and maturation of pro-inflammatory cytokines such as interleukin-1β (IL-1β) and interleukin-18 (IL-18). These cytokines then act on various cell types within the vasculature. On one hand, they stimulate endothelial cells to express adhesion molecules, which attract immune cells and promote their recruitment to the vessel wall. This local inflammation disrupts the normal anticoagulant properties of the endothelium. On the other hand, the released cytokines can directly or indirectly enhance the activation of coagulation factors. For example, they can upregulate the expression of TF, a key initiator of the extrinsic coagulation pathway. Moreover, inflammasome activation also has a direct impact on platelets. It can promote platelet aggregation by increasing the sensitivity of platelets to activating stimuli, leading to the formation of platelet aggregates. Collectively, these processes driven by inflammasome activation significantly contribute to the formation of thrombosis.

*MCC950:* MCC950 is a selective inhibitor of NLRP3 [194]. In a rat model of DVT, MCC950 injection effectively mitigated DVT-induced venous injury, demonstrating its therapeutic potential [195]. However, the pharmacokinetic and toxicological properties of MCC950 have limited clinical use. For example, high doses of MCC950 may produce adverse effects, leading to its suspension in phase II clinical trials [196]. In the future, to improve the safety of MCC950 in clinical applications, efforts should be made to optimize its metabolic and toxicological characteristics. By enhancing its metabolism, the drug can be cleared from the body more efficiently, reducing the risk of accumulation and potential toxicity. Meanwhile, minimizing its toxicological impact can directly lower the occurrence of adverse effects during treatment.

*Hirudin:* Hirudin, a secretion from the leech salivary gland, has anti-inflammatory properties. In the mouse MCA occlusion/reperfusion model, hirudin significantly reduced the extent of cerebral infarction and significantly improved motor deficits [194]. Although Hirudin has shown advantages in anticoagulant therapy, its use is still limited to some extent. For example, its short half-life (about 25 minutes) requires frequent administration. In addition, the immunogenicity of Hirudin may cause severe allergic reactions. Future studies should further optimize the pharmacokinetic properties of Hirudin and its derivatives and explore its potential applications in other diseases.

*Canakinumab:* The CANTOS trial showed that canakinumab, an anti-IL-1β antibody, reduced the recurrence of myocardial infarction (MI), particularly in patients with a previous MI and elevated plasma levels of high-sensitivity C-reactive protein [197]. The safety profile of canakinumab was generally good. The most common adverse events were injection site reactions, headache, and muscle pain. However, its use is associated with an increased risk of serious infections, especially at high doses or with prolonged treatment. In addition, it has been suggested that canakinumab may mask symptoms of infection, so close monitoring of the patient's health status is necessary during treatment.

### 2.3.4 Targeting platelet-neutrophil interactions

The interaction between platelets and neutrophils plays a crucial role in promoting thrombus formation. This intricate process involves a series of well-coordinated steps. Firstly, platelets and neutrophils are able to adhere to each other through specific adhesion molecules. For instance, P-selectin expressed on activated platelets can bind to P-selectin glycoprotein ligand-1 (PSGL-1) on neutrophils. This binding event not only physically links the two cell types but also initiates intracellular signaling cascades within both platelets and neutrophils. Once the connection is established, these cells start to release a variety of inflammatory mediators. Platelets secrete substances such as platelet-derived growth factor (PDGF), serotonin, and TxA2. Neutrophils, on the other hand, release cytokines like IL-1β and tumor necrosis factor-α (TNF-α), as well as chemokines such as interleukin-8 (IL-8). These inflammatory mediators create a pro-inflammatory microenvironment at the site of potential thrombus formation. In addition to inflammatory mediators, platelets and neutrophils also release TFs. TFs are transmembrane glycoproteins that play a key role in initiating the extrinsic coagulation pathway. When exposed to the blood, TFs form a complex with factor VII/VIIa, which then activates FX. This activation sets off a cascade of enzymatic reactions that ultimately lead to the conversion of prothrombin to thrombin. Thrombin, in turn, catalyzes the conversion of fibrinogen to fibrin, resulting in the formation of a fibrin meshwork that traps blood cells and platelets, promoting the development of a stable thrombus. In conclusion, the interaction between platelets and neutrophils, mediated by adhesion molecules, and followed by the release of inflammatory mediators and TFs, effectively stimulates the coagulation reaction and eventually culminates in thrombosis.

P-selectin Inhibitors: P-selectin plays a critical role in the formation of platelet-leukocyte aggregates [198]. It is involved in both inflammation and thrombosis, and in both agonist- and shear- associated platelet aggregation, thereby contributing to thrombus growth and stabilization [199].

*THCMA:* THCMA is a P-selectin inhibitor with a 100 nm nano-ring structure. When administered orally, THCMA was 100 times more effective than the investigational drug PSI-697 in inhibiting both AT and VT [200]. Since THCMA has not yet entered the stage of human clinical trials, the effects of its long-term use on the cardiovascular system still need to be further studied. Future studies should focus on the dose dependence and mechanism of action of THCMA as well as the results of human clinical trials to fully evaluate its clinical application value.

*Crizanlizumab:* Crizanlizumab works by binding to P-selectin and blocking it from interacting with PSGL-1. The role of P-selectin in COVID-19 pulmonary thrombosis has been demonstrated, but further studies are needed to determine whether crizanlizumab can have a preventive effect [201]. However, the potential risks still need to be paid attention to. For example, in one study, there were three deaths in the crizanlizumab group, but these deaths were not considered to be related to the study drug. In addition, crizanlizumab may interfere with platelet counts and therefore require regular monitoring.

**ICAM-1 Inhibitors**: ICAM-1 plays a role in regulating platelet-neutrophil interactions. Inhibiting ICAM-1 function has the potential to reduce thrombosis by disrupting these interactions.

*Enlimomab (R6.5):* Enlimomab is an anti-ICAM-1 monoclonal antibody whose role in stroke treatment is in clinical trials. However, the lack of significant treatment effects in some studies, along with safety concerns, led to the discontinuation of further development of enlimomab [202]. Although enlimomab has not been successful in the treatment of ischemic stroke, its potential anti-inflammatory effect still deserves further exploration. For example, researchers could consider developing humanized or chimeric antibodies to reduce immunogenicity and improve patient tolerance. In addition, the combination of other immunomodulatory strategies, such as the combination of thrombolytic drugs or anti-inflammatory drugs, may provide new ideas for improving the treatment effect.

*LY-2157299:* LY-2157299, a small-molecule ICAM-1 inhibitor, has demonstrated significant anti-inflammatory and antithrombotic effects in animal models. In vivo, LY-2157299 protects mice from endotoxemia by inhibiting ICAM-1. Although LY-2157299 did not show significant cardiotoxicity, its effects on cardiac electrophysiological parameters still need to be continuously monitored. For example, in one study, changes in heart rate occurred in some patients, although no prolongation of the QTc interval was observed [203].

*Tremacamra:* Tremacamra is an anti-ICAM-1 monoclonal antibody. However, in patients with non-ST-elevated MI, tremacamra did not reduce myocardial injury [204]. Currently, the optimal mode of administration of tremacamra (e.g., by inhalation, oral administration, or nasal spray) is unknown, which could affect its clinical use.

Studies have shown that inflammation plays an important role in the process of thrombosis. In addition, activated platelets are able to promote inflammatory responses and release inflammatory factors, which further aggravate thrombosis. Anti-inflammatory drugs have shown potential in the treatment of thrombotic diseases, such as atherosclerotic thrombosis and venous thromboembolism, but the complex mechanisms of inflammation and thrombosis still need to be further explored. Future research should focus on precise targeted therapy, combination strategies, the application of inflammatory markers, and the development of new anti-inflammatory drugs to achieve more efficient and safer treatment effects.

## 3. New approaches for the treatment of thrombosis: nanomedicine delivery

**Figure 7.**
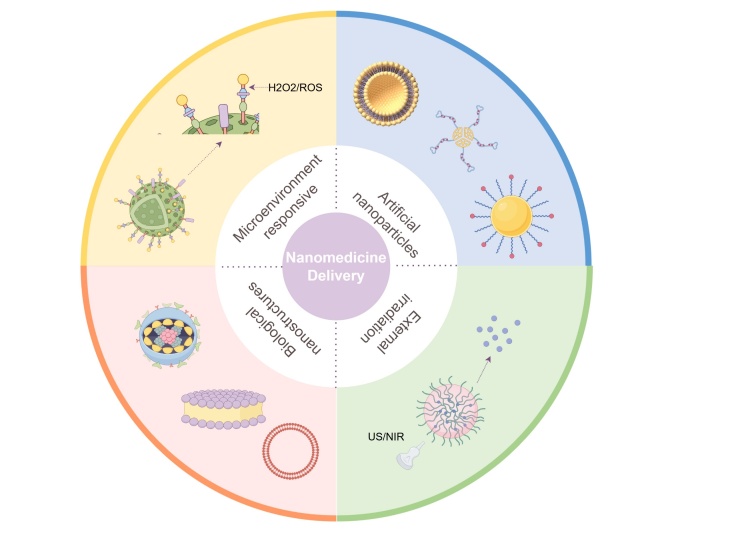
**Nanodrug Delivery Systems**. According to the structure and drug release methods of nanoparticles, they are divided into artificial nanoparticles, microenvironment responsive release, biomimetic nanoparticles and external irradiation treatment.

The major disadvantages of fibrinolytic therapy include treatment failure, which can result from ineffectiveness or re-thrombosis due to persistent vascular lesions and a hypercoagulable state [205]. The integration of nanomedicine with thrombolytic therapy has gained significant attention, driven by advancements in nanotechnology. Nanodrug-delivered "thrombo-disrupting" agents are expected to act locally at the thrombus site, providing highly effective recanalization rates while reducing the bleeding complications associated with systemic intravenous administration. Currently, there are four main categories of nanomedicine for the treatment of thrombosis, classified by their structure or mode of action represented in Figure 7 [206].

### 3.1 Artificial nanoparticles-based drug delivery

Three key factors should be considered when designing an artificial nanoparticle targeted delivery system: 1) The material of the nanomedicine should be non-toxic and have a shape and modifications that make it more accessible to the thrombus site; 2) The thrombolytic agent should remain encapsulated and not be released before the nanocarrier has accumulated at the target site; 3) Whenever possible, multifunctional nanoparticles should be loaded or modified with contrast agents to enable monitoring of the treatment process [206].

### 3.1.1 Nano-drug delivery system based on liposomes

A liposome is a spherical carrier consisting of a phospholipid bilayer [207]. They are ideal for encapsulating thrombolytic drugs due to their ease of surface modification, good biocompatibility, low toxicity, and simple preparation, making them a top choice for nanomedicine design [208]. Liposomes for small molecule drug delivery have been approved by the FDA [207]. Encapsulating fibrinogen activators in liposomes is commonly used for clot-specific drug delivery, enhancing drug half-life and reducing hemorrhagic side effects.

Liposomes can be engineered to aggregate at thrombus sites by modifying their surface with site-specific targeting ligands. For example, targeting the platelet membrane (PM) glycoprotein GPⅡb/Ⅲa that is significantly overexpressed in thrombosis [209].

**RGD Peptides**: RGD peptides are a group of short peptides containing Arg-Gly-Asp, a sequence recognized by most integrins for ligand binding. Modifying liposomes with RGD peptides can enhance their ability to target integrins directly. Linear RGD peptides often have short circulating half-lives. Stability can be improved by converting the linear peptide structure into a cyclic form.

As early as 2011, Vaidya et al. found that liposomes modified by cyclic RGD (cRGD) peptide were significantly aggregated at the thrombus site in rat [210].

In the following years, studies demonstrated that cRGD liposome-encapsulated eptifibatide had enhanced antiplatelet activity in vitro [211]. Further studies showed that urokinase encapsulated in cRGD-functionalized liposomes reduced 75% urokinase dose in the mouse mesenteric thrombosis model, but achieved the same thrombolytic effect as free urokinase [208]. Studies in the past two years have found that cRGD-modified liposomes also show obvious therapeutic advantages in acute pulmonary microthromboembolism and pulmonary hypertension disease models [212, 213].

**Hyperechoic Liposomes (ELIPs)**: ELIPs are bilayer phospholipid vesicles containing gaseous microvesicles. It was shown that plasmin was encapsulated in ELIPs and released only upon US-triggered clot activation, preventing rapid inhibition by endogenous plasmin inhibitors [214].

Nanoliposome encapsulated urokinase containing ironoxide, perfluorohexane (PFH) enables near-infrared (NIR) and US-triggered thrombolysis, as well as multimodal imaging for diagnostic purposes [215]. In addition, ultrasound-responsive nano-liposome capsules encapsulated with urokinase were injected in situ through intravenous catheters in rabbits with PE, showing the therapeutic potential of acute PE [216].

### 3.1.2 Polymer-based nanodrug delivery system

Polymers can form nanospheres and nanocapsules, which are used as nanocarriers. These nanocarriers are primarily made from two types of polymers: natural and synthetic. Natural polymers include polysaccharides such as hyaluronic acid, alginate, and chitosan, as well as proteins like gelatin and albumin. Synthetic polymers include polycaprolactone (PCL), polylactic acid (PLA), and polyamide-amine (PAMAM) [217]. Both natural and synthetic polymers have their advantages and disadvantages. Natural polymers offer good biocompatibility but can exhibit significant batch-to-batch variability. Synthetic polymers, on the other hand, are highly pure and reproducible. The coagulant binds to the surface of the nanoparticles.

**Chitosan**: Chitosan and its derivatives have excellent biocompatibility, which are ideal materials for the sustained release of thrombolytic drugs. It has been shown that chitosan-loaded uPA exhibits higher thrombolytic efficiency than free uPA in thrombin-induced VT in rabbits [218]. Furthermore, intravenous administration of ASA-RGD-CS@TPP greatly enhanced anti-thrombotic efficacy with reduced blood loss compared to aspirin alone in a mouse model [219].

**Gelatin**: The presence of acidic and basic functional groups on the gelatin peptide chain gives it amphoteric polyelectrolyte properties, thus allowing chemical modification by derivatization. As a natural protein hydrolysate, gelatin has good biocompatibility and biodegradability. Coupling the CREKA peptide to the gelatin surface, followed by phagocytosis by macrophages, creates a bionic system. Upon LIFU irradiation, NPs are released for deposition at the site of the thrombus [220].

**PLGA**: PLGA nanoparticles are submicron (< 1 μm) polymer colloidal particles made from a block copolymer of glycolic acid (GA) and lactic acid (LA). Their degradation products (water and carbon dioxide) are eliminated through the Krebs cycle, minimizing irritation and side effects. Chemical modification of PLGA surface can further enhance the effect, such as the use of methoxy-polyethylene glycol (mPEG)-PLGA nanoparticles to encapsulates streptokinase, which significantly prolongs the drug circulation time (up to 120 minutes) and improves the pharmacokinetic properties [221]. Zhang et al. conducted an innovative study to design PB-PFP@PC with Prussian blue (PB), attaching CREKA as a delivery carrier shell to PLGA. A novel drug-free dual thrombolysis strategy was achieved by combining PTT with ODV under NIR laser irradiation [222]. More studies have shown that curcumin (Cur) and PFP as the core components, PLGA as a substrate, coated with PM, to synthesize a nanomaterial (Cur-PFP@PC). The system is particularly suitable for pregnancy VT [223].

### 3.1.3 Inorganic nanoparticles

Inorganic nanoparticles (INPs) are synthesized from inorganic particles combined with biodegradable polycations. Typical examples of INPs include metals, metal oxides, carbon materials, and magnetic nanoparticles (MNPs), which are often composed of superparamagnetic iron oxide nanoparticles (SPION).

**MNPs**: Ferrite nanoparticles are the most studied MNPs, and their magnetic properties can be significantly enhanced by clustering individual superparamagnetic MNPs into magnetic beads. MNPs can be selectively attached to functional molecules and guided to target sites using an external magnetic field from electromagnets or permanent magnets. To prevent aggregation and minimize particle interactions with the surrounding environment, surface coatings such as surfactants, silica, organosilicon, or phosphoric acid derivatives are often used to increase stability in solutions. The study of MNPs started early. More than a decade ago, it was found that encapsulating urokinase in MNPs enhanced thrombolytic effect by 5 times without increasing bleeding side effects [224]. The role of MNP in other related disease models has been studied. Loading biodegradable superparamagnetic MNPS into endothelial cells and guiding them into the stent under a magnetic field to restore blood flow can alleviate in-stent stenosis [225]. In recent years, Banik et al. developed a dual-targeted nanoparticle library of encapsulated iron oxide nanoparticles and determined its MRI contrast enhancement ability for imaging mouse heart and aorta [226]. More recently, a nanoparticle platform (MFe_2_O_4_-ZnDPA NPs) has been developed to enhance aneurysm-associated thrombus detection and aneurysm treatment, which can target the thrombus through the specific interaction of ZnDPA with phosphatidylserine of activated platelets in the thrombus. In a rabbit common carotid artery aneurysm model, it was shown to accumulate within aneurysm-associated thrombi [227]. In a rabbit model, thrombolytic efficacy was amplified by using rtPA-modified magnetite nanoparticles (rtPA-Fe_3_O_4_NPs) below the clinical rtPA drug concentration [228]. Recently, a ferromagnetic liquid robot (FMLR) consisting of Fe_3_O_4_-MNP and dimethylsilicone oil has also been developed, which is capable of navigating narrow and complex blood vessels through shape deformation under an external magnetic field, showing the potential for remote robotic neurointerventional therapy [229].

**Gold Nanoparticles (AuNPs)**: Gold (Au) is chemically stable, and it exhibits unique photoelectric, physical and chemical properties at the nanoscale, and has good biocompatibility. The delivery of thrombolytic drug urokinase by gold nanoparticles showed a significant photothermal effect, resulting in rapid release of the drug under NIR irradiation [230]. Chang et al. developed Si-AuNRs with multifunctional features such as thrombolytically targeted drug delivery, which improves imaging and thrombolytic efficiency while reducing the risk of bleeding [231]. The targeting of small AuNPs to neutrophils has been demonstrated in APS mice and is expected to be applied to the treatment of antiphospholipid syndrome [232]. A recent study has prepared a nano-coupling system AuNP-Cl that can target collagen and early thrombosis, and its binding to the collagen surface has been observed. In addition, dark field microscopy has confirmed that AuNP-Cl has good imaging ability, which will contribute to the development of multifunctional antithrombotic reagents [233].

### 3.2 Microenvironment responsive drug delivery system

An effective responsive delivery system should incorporate factors that promote the high accumulation of nanomedicine in thrombotic tissue. Additionally, the design of such systems should be closely aligned with the thrombotic microenvironment, ensuring that therapeutic agents are released specifically within the thrombus without affecting normal tissues [206].

### 3.2.1 H_2_O_2_ response

H_2_O_2_ plays a role in signal transduction pathways within the vascular system [234], and its levels are significantly elevated at the thrombus site. H_2_O_2_-responsive nanomedicine has been developed to enable targeted release of therapeutic agents specifically at the thrombus location. Due to the presence of large amounts of fibrin and H_2_O_2_ at the thrombus site, a tirofiban-delivery nanocarrier called T-RBC-DTC conjugates tirofiban to dextran via a H_2_O_2_-sensitive phenyl boric acid bond, which enhances the efficacy of tirofiban. Similarly, argatriban loaded H_2_O_2_-responsive nanoparticles (PNPArg) significantly inhibited thrombosis and reduced H_2_O_2_ level in a mouse thrombosis model [231]. In another structure, the boric acid linker dimerizes two at RA molecules. The linker is specifically cleaved upon exposure to H_2_O_2_, releasing the anti-inflammatory oxybenzyl alcohol and scavenging H_2_O_2_, which significantly inhibits thrombosis[235]. H_2_O_2_ triggered nanoparticles (CyBA/PFM NPs) can be activated in response to H_2_O_2_. In the AT model, CyBA/PFM NPs can remove ROS and inhibit thrombosis [236].

### 3.2.2 ROS response

ROS are upregulated at thrombus sites [237], leading to endothelial dysfunction and platelet activation, which promotes thrombus extension [234]. Controlled drug release can also be triggered by ROS levels in the thrombus microenvironment. ROS-responsive amphiphilic chimeric drug delivery platforms have been developed. Dual drug-loaded nanoparticles show significant therapeutic effects in thrombosis model [238].

### 3.3 Drug delivery systems using biological nanostructures

Cell membrane nanoparticles derived from natural cells are significant in nanomedicine delivery due to their advantages, including high biocompatibility, extended circulation time, and the potential for genetic engineering modifications [239, 240].

### 3.3.1 Platelet membrane

Nanoparticles based on PM can facilitate immune escape in vivo and evade macrophage clearance, making them valuable for use in thrombosis therapy [241]. It has been shown that nanoclusters encapsulated in PMs can target the wall of damaged arteries prone to restenosis, thus solving the problem of restenosis after angioplasty [242]. In mice with PE and mesenteric artery thrombosis, encapsulated rt-PA nanoparticles aggregated at the clot site and markedly enhanced thrombolytic activity [320]. More studies have shown that by extruding PMs and inserting Annexin V into liposomes, APLT-PA can be prepared, which leads to significant improvements in thrombolytic and neurological functions in AIS mice [243]. In addition, it has been reported that a thrombus microenvironment responsive nano-capsules coated with PMs can adhere to damaged endothelial cells and thus treat AMI [244]. Moreover, a melanin nanoplatform based on the porphyrin covalent organic framework can mimic platelets and deliver hirudin to the VT site to achieve noninvasive thrombolysis and effective anticoagulation [245].Similarly, in the treatment of VT, another platelet nano platform for targeted drug treatment of DVT can also effectively eliminate DVT lesions, and the conventional dose can generally achieve the ideal therapeutic effect [246].

### 3.3.2 Erythrocyte membrane

Red blood cell-camouflaged nanoparticles (RMPNP) are a system designed for delivering drugs, enzymes, peptides, antigens, and other substances in vivo, utilizing red blood cells or RM nanovesicles as carriers. RMPNPs offer a number of advantages, including excellent biocompatibility, prolonged circulation time and improved targeting capabilities. Because tirofiban could potentially impair receptors on PMs and thus affect targeting, Zhao et al. constructed erythrocyte membrane-coated nanoparticles (T-RBC-DTC NPs). These nanoparticles showed significantly enhanced antithrombotic activity [247]. Moreover, extended circulation half-life (t^1^/_2_ = 3.28 h) of erythrocyte membrane-concealed nano-capsules (USIO/urokinase @EM) for intravenous and arterial thrombolysis was achieved by the application of RM coating [248].

### 3.4 External irradiation treatment

Treatment strategies utilizing external irradiation combined with mechanical stress, hyperthermia, and ROS response, along with *in vivo* imaging, enable targeted irradiation solely at the thrombus site, thereby minimizing damage to surrounding normal tissues [249].

### 3.4.1 Ultrasound

US typically influences the physical, chemical, or thermal stability of drug delivery systems through its mechanical and thermal effects. This interaction facilitates the release or activation of drug units, enabling controlled drug-mediated treatment of diseases [250]. The drug release of ELIPs described above is by this principle. PPrC nanoparticles loaded with PFH and rtPA, when combined with the mechanical activation of thrombolytic agents, become a reliable method for thrombus monitoring and treatment. In vivo experiments have shown that these nanoparticles can monitor thrombosis, exhibit strong thrombolytic effects, and significantly reduce adverse bleeding reactions [251]. In addition, porous magnetic microbubble platforms have been developed to address issues such as low tPA bioavailability. This helps to maintain tPA activity while circulating and improves drug penetration into the thrombus. In VT mice, residual thrombus was reduced by 67.5% [252]. In addition, a C-shaped magnetic actuation system can further enhance thrombolysis by allowing tPA to reach the thrombus through interrupted blood flow by movement of the MNP [253]. The multifunctional pathology mapping therapy diagnostic nanoplatform (MPmTN) identifies types of weak plaques and determines more accurate treatment strategies for atherosclerosis [254].

### 3.4.2 Near infrared light

Photo-controlled drug delivery systems are prepared from materials containing photosensitive groups. To achieve non-invasive, demand and sustained anti-thrombotic treatment, a study was conducted in which NIR light activates the temperature-sensitive TRPV1 channel via photothermal effects, triggering a synthesized pathway to secrete uPA. It has shown a significantly enhanced thrombolytic effect in the mouse tail thrombosis model [255]. A targeted photothermal technique uses macrophages loaded with NIR polypyrrole-poly-ethylenimine nanocomposites to prevent and reduce fibrin clots [256]. Similarly, significant fibrin clot clearance can also be achieved by targeting inflammatory endothelial cells/thrombus sites with high acid/P-selectin expression GPS-ppy-H NPs and NIR irradiation [257]. Recently, Song et al. created a polymer nanoplatform with integrated thrombus detection and antithrombotic activity based on NIR-II photoacoustic imaging, which demonstrated rapid and effective thrombus removal in AT models [258]. This year, a novel dual-mode strategy combining US and NIR demonstrated aggregation and penetration at thrombus sites in vitro and in vivo, thus establishing a synergistic approach to directed imaging and thrombolytic therapy under the combined effects of US and NIR [259].

The precise targeting of the thrombus site by specific peptides (such as cRGD peptide, CREKA peptide, CLT peptide) or biological materials (such as MNPs, PM nano-sponges) has significantly improved the therapeutic effect and reduced the risk of bleeding. For example, cRGD peptides are used for early thrombus detection and thrombolysis kinetic monitoring because of their high affinity for GPIb/IIa receptors. In addition, MNPs combined with high shear stress allow rapid thrombus removal. In conclusion, nanomedicine has shown great potential in the field of antithrombotic therapy, which significantly improves the therapeutic effect through targeting, intelligent design and multifunctional strategies. However, its clinical translation still needs to overcome challenges in technology, safety and metabolic toxicity. Future research should focus on optimizing the design of nanomedicine, improving the ability of clinical translation, and combining multimodal treatment strategies to achieve more efficient and safer antithrombotic regimens.

## 4. Conclusions and perspectives

Thrombosis is a leading cause of disability and death. There are distinct differences in the risk factors and formation mechanisms between AT and VT, which subsequently influence their composition and, in turn, affect the choice of treatment methods.

There are two primary approaches to treating thrombosis: surgical treatment and pharmacological treatment. Surgical interventions are mainly reserved for severely ill patients, while drug therapy remains the primary method for most patients. Currently, drugs for thrombosis are primarily classified into three categories based on their targets: antiplatelet agents, anticoagulants, and fibrinolytic agents. Many classical antithrombotic drugs (such as aspirin, clopidogrel, warfarin, etc.) may increase the risk of bleeding. For example, aspirin inhibits platelet aggregation by irreversibly inhibiting cyclooxygenase (COX), leading to a reduction in the production of TxA2, which is a bioactive substance with strong pro-platelet aggregation and vasoconstrictive effects, but it also reduces the coagulation mechanism and predisposes to bleeding. In addition, as a vitamin K antagonist, warfarin requires regular monitoring of the prothrombin time to avoid bleeding due to excessive anticoagulation. Newer antiplatelet agents (e.g., prasugrel and ticagrelor) have greater efficacy than clopidogrel but are associated with a higher risk of bleeding. In addition, some classical antithrombotic drugs have limited efficacy in specific patient groups. For example, aspirin has limited efficacy in preventing cardiovascular disease in healthy people and cannot be used to prevent ischemic stroke. Moreover, classical antithrombotic agents may cause a variety of side effects. For example, aspirin may irritate gastric mucosa, causing ulcers or bleeding; Warfarin may cause liver injury. These side effects limit the drug's applicable population and long-term safety [260]. Classical antithrombotic drugs need to be improved in terms of drug interaction and compliance [261], difficulty in drug dose adjustment, and treatment cost.

In recent years, great progress has been made in the research of antithrombotic drugs. New antiplatelet drugs such as P2Y12 receptor antagonists (clopidogrel, ticagrelor and prasugrel), which inhibit platelet aggregation by blocking ADP receptors, have become the mainstream of antiplatelet therapy [262, 263]. DOACs, such as rivaroxaban and apixaban, have received considerable attention for their low bleeding risk and high efficacy. By inhibiting prothrombin enzyme, these drugs effectively reduce thrombosis while reducing the risk of bleeding. New thrombolytic drugs, such as recombinant hirudin and Fangpadalu, can rapidly dissolve thrombosis by activating plasminogen to promote the generation of plasmin. In addition, the use of nanotechnology to optimize the local concentration and release characteristics of thrombolytic drugs has improved the efficacy and reduced the risk of bleeding [264]. More importantly, the exploration of targeted therapy with new targets is ongoing. Contact pathway activators (such as NETs and polyphosphates) are thought to more specifically inhibit thrombosis while reducing bleeding risk [265, 266]. Shanghai Institute of Material Medicine has solved the three-dimensional structure of the P2Y12 receptor, which provides key clues for the development of a new generation of antithrombotic drugs. Smart antithrombotic nanodrugs are also being developed in parallel. Intelligent delivery systems that enable precise drug delivery by light control or synthetic complementary sequences could further improve efficacy and reduce side effects. In addition, image-guided therapy combines nanotechnology and imaging technology to achieve accurate localization and treatment of thrombus. The research and development of antithrombotic drugs is moving towards more efficient and safer options and combined with new technologies such as nanotechnology and intelligent delivery systems, it provides more clinical options. However, how to balance efficacy with bleeding risk remains an important topic for future research.

This article summarizes new antithrombotic drug targets and the corresponding drugs or formulations currently under study or in clinical trials, based on the roles of platelets, the coagulation pathway, and inflammation in thrombosis. Given the excellent performance of nanomedicine in reducing bleeding side effects and extending drug circulation time, we also highlight methods for novel packaging or targeted delivery of drugs for thrombosis treatment that are currently being investigated. These targets, drug formulations, and delivery methods can improve the shortcomings of current drugs for the treatment of thrombosis, such as side effects, and show considerable advantages in vivo or in vitro experiments. In the future, a specific drug or preparation can be modified, or a larger clinical trial can be conducted to improve its clinical value.

## Supplementary Materials

The Supplementary data can be found online at: www.aginganddisease.org/EN/10.14336/AD.2024.1688.
