# Supplementary material for: Emerging Targets, Novel Directions, and Innovative Approaches in Thrombosis Therapy
Source: Aging Dis. 2025 Mar 11;17(2):812–48. doi: 10.14336/AD.2024.1688 (PMC12834416; doi:10.14336/AD.2024.1688)
Supplement: Supplementary file 1 — The Supplementary data can be found online at: www.aginganddisease.org/EN/10.14336/AD.2024.1688. [file AD-17-2-812-s.pdf]

SUPPLEMENTARY DATA

Emerging Targets, Novel Directions, and Innovative Approaches in Thrombosis Therapy

Weiyue Zhang, Baoqing Pei, Yifan Zhou, Hui Li, Wei Ma, Bing Zhou, Chen Zhou, Huimin Jiang, Xunming Ji

© 2025. Zhang W et al. Published online at http://www.aginganddisease.org/EN/10.14336/AD.2024.1668

SUPPLEMENTARY DATA

Supplementary Table 1. Antiplatelet drugs currently used in clinical practice

Category	Name	Mechanism
Cyclooxygenase (COX) inhibitors	Aspirin [1]	The most commonly used antiplatelet drug, reduces the production of thromboxane A2 by inhibiting COX-1.
P2Y12 receptor antagonist	Clopidogrel [2]	It requires hepatic metabolic activation and inhibits P2Y12 receptors.
	Prasugrel [3]	The onset of action is faster and the anti-platelet effect is stronger
	Ticagrelor [4]	Reversible P2Y12 receptor antagonist without metabolic activation
	Cangrelor [5]	Intravenous injection, rapid onset of action, suitable for emergency situations
Glycoprotein IIb/IIIa receptor antagonists	Abciximab [6]	Monoclonal antibodies, administered intravenously, are used for acute coronary syndromes or PCI
	Tirofiban [7]	Small non-peptide antagonists, intravenous
	Eptifibatide [8]	Cyclic peptide antagonists, IV
Phosphodiesterase inhibitor	Dipyridamole [9]	It enhances the antiplatelet effect by inhibiting phosphodiesterase and adenosine reuptake and is often used in combination with aspirin
	Cilostazol [10]	Phosphodiesterase III inhibitors, which have antiplatelet and vasodilator effects, are mainly used in peripheral arterial disease.
Thromboxane A2 receptor antagonists	Terutroban [11]	It directly inhibits thromboxane A2 receptors and reduces platelet aggregation
Protease activated receptor-1 (PAR-1) antagonist	Vorapaxar [12]	Inhibition of PAR-1, which reduces thrombosis, is used in patients with a history of myocardial infarction or peripheral arterial disease

Supplementary Table 2. Anticoagulation currently used in clinical practice

Category	Name	Mechanism
Vitamin K antagonists (VKAs)	Warfarin [13]	By inhibiting vitamin K-dependent synthesis of coagulation factors (II, VII, IX, and X), INR should be monitored regularly.
Direct oral anticoagulants (DOACs)	Dabigatran [14]	Direct inhibition of thrombin
	Rivaroxaban [15]	Direct inhibition of FXa
	Apixaban [16]	Direct inhibition of FXa
	Edoxaban [17]	Direct inhibition of FXa
Heparins	Unfractionated Heparin, (UFH) [18]	By enhancing the activity of antithrombin III and inhibiting multiple coagulation factors, APTT needs to be monitored
	Low Molecular Weight Heparin, (LMWH) such as, Enoxaparin and Dalteparin [19]	The mechanism of action is similar to that of unfractionated heparin, but it has a longer half-life and is more convenient to use.
Antithrombin III activator	Fondaparinux [20]	Selective inhibition of FXa is used to prevent and treat VT
Direct thrombin inhibitor	Bivalirudin [21]	It directly inhibits thrombin and is mainly used for anticoagulation during PCI
Other anticoagulants	Argatroban [22]	Direct thrombin inhibitors for heparin-induced thrombocytopenia (HIT)
	Danaparoid [23]	It is used for anticoagulant therapy in patients with HIT
Novel oral anticoagulants (NOACs)	Betrixaban [24]	Direct inhibition of FXa for the prevention of venous thromboembolism

© 2025. Zhang W et al. Published online at http://www.aginganddisease.org/EN/10.14336/AD.2024.1668

SUPPLEMENTARY DATA

Supplementary table 3: Novel antiplatelet agents in preclinical or clinical development

Target	Name	Class	Phase of development	Route of administration
ADP-receptor	***Elinogrel***	Small molecule	Phase II	Oral
	MRS2500	Small molecule	Preclinical	Intravenous
	BMS-884775	Small molecule	Preclinical	Oral
	GLS-409	Small molecule	Preclinical	Intravenous
	Selatogrel	Small molecule	Phase II/III	Subcutaneous
	SAR216471	Small molecule	Preclinical	Oral
	AZD1283	Small molecule	Preclinical	Oral
PAR	***Atopaxar***	Small molecule	Phase II	Oral
	SCH 79797	Small molecule	Preclinical	Intravenous
	FR 171113	Small molecule	Preclinical	Subcutaneous
	PZ-128	Small molecule	Phase II	Intravenous
	BMS-986120	Small molecule	Phase I	Oral
	BMS-986141	Small molecule	Phase IIa	Oral
	SCH-28	Small molecule	Preclinical	Intravenous
GPVI	***ACT017***	Antibody	Phases II/III	Intravenous
	Revacept	Fusion protein	Phase II	Intravenous
	Losartan	Small molecule	-	Oral
	Anfibatide	Small molecule	Phase II	Intravenous
	Honokiol	Small molecule	Phase III	-
	SAR264565	Antibody	Preclinical	-
	GPVI-CD39	Fusion protein	Preclinical	Intravenous
	Troα6/Troα10	Small molecule	Preclinical	Oral
GPIb-VWF interaction	***h6B4-Fab***	Antibody	Preclinical	Intravenous
	TaSER	Fusion protein	Preclinical	-
	AJW200	Antibody	Preclinical	Intravenous
	82D6A3	Antibody	Preclinical	Intravenous
	ARC1779	Aptamer	Phase I	Intravenous
	ARC15105	DNA aptamer	Preclinical	Subcutaneous
	BT200	DNA aptamer	Phase II	Subcutaneous
	TAGX-0004	DNA aptamer	Preclinical	-
	ALX-0081	Nanobodies	Preclinical	Intravenous
	Caplacizumab	Immunoglobulin	Phase III	Intravenous
CLEC-2	***2CP***	Small molecule	Preclinical	Intravenous
5-HT/receptor interactions	***MCI-9042***	Small molecule	-	Oral
	Temanogrel	Small molecule	Preclinical	Oral
	5HT2ARAb	Antibody	Preclinical	-
adenosine A2A and A2B receptors	***NECA***	Small molecule	Preclinical	-
αIIbβ3 integrin	***RUC-4***	Small molecule	Phase III	Oral
PI3Kβ	***TGX-221***	Small molecule	Preclinical	Intravenous
	AZD6482	Small molecule	Phase IIa	Intravenous
	MIPS-9922	Small molecule	Preclinical	Intravenous
Syk	***Fostamatinib***	Small molecule	Preclinical	Oral
	BI1002494	Small molecule	Preclinical	Oral
12-LOX	***ML355***	Small molecule	Preclinical	Oral
BTK	***AB-95-LH34***	Small molecule	Preclinical	-
	Acalabrutinib	Small molecule	Preclinical	Oral
	Evobrutinib	Small molecule	Phase II	Oral
	Ibrutinib	Small molecule	Preclinical	Oral
	Remibrutinib	Small molecule	Phase II	Oral
PDI	***RL90***	Small molecule	Preclinical	Intravenous
	Quercetin-3-rutinoside	Flavonoids	Preclinical	Intravenous
	Isoquercetin	Flavonoids	Phase II/III	Oral
	Myricetin	Flavonoids	Preclinical	Intravenous

© 2025. Zhang W et al. Published online at http://www.aginganddisease.org/EN/10.14336/AD.2024.1668

**PDK1**	***BX795***	Small molecule	Preclinical	Intravenous
**CK2**	***CX-4945***	Small molecule	Preclinical	Intravenous
	TBCA	Small molecule	Preclinical	Intravenous
**SGK1**	***GSK-650394***	Small molecule	Preclinical	Intravenous
**Annexin A7**	***ABO***	Small molecule	Preclinical	Intravenous
**PDK**	***DCA***	Small molecule	Preclinical	Intraperitoneal
**PKM2**	***ML265***	Small molecule	Preclinical	-
	DASA	Small molecule	Preclinical	Intravenous
**β-Oxidation of fatty acids**	***Etomoxir***	Small molecule	-	-
**Glutamine oxidation**	***Azaserine***	Small molecule	-	-

Supplementary table 4: Novel anticoagulant agents in preclinical or clinical development

**Target**	**Name**	**Class**	**Phase of development**	**Route of administration**
**FXII**	***Infestin-4***	Natural inhibitors	Preclinical	Intravenous
	Ir-CPI	Natural inhibitors	Preclinical	Intravenous
**FXI**	***AB023***	Antibody	Phase II	-
	MAA868	Antibody	Phase I	Subcutaneous
	BAY1213790	Antibody	Phase II	Intravenous
	BMS-986177	Small molecule	Phase II	-
	ONO-7684	Small molecule	-	Oral
	EP-7041	Small molecule	-	Intravenous
	BAY 2433334	Small molecule	Phase III	Oral
	Fasxiator	Natural inhibitors	-	-
	IONIS-FXI-Rx	ASOs	Phase III	Subcutaneous

References

[1] Fuster V, Sweeny JM (2011). Aspirin: a historical and contemporary therapeutic overview. Circulation, 123:768-778.

[2] Kuszynski DS, Lauver DA (2022). Pleiotropic effects of clopidogrel. Purinergic Signal, 18:253-265.

[3] Cagnazzo F, Perrini P, Lefevre PH, Gascou G, Dargazanli C, Riquelme C*, et al.* (2019). Comparison of Prasugrel and Clopidogrel Used as Antiplatelet Medication for Endovascular Treatment of Unruptured Intracranial Aneurysms: A Meta-Analysis. AJNR Am J Neuroradiol, 40:681-686.

[4] D'Amario D, Galli M, Restivo A, Canonico F, Vergallo R, Migliaro S*, et al.* (2024). Ticagrelor enhances the cardioprotective effects of ischemic preconditioning in stable patients undergoing percutaneous coronary intervention: the TAPER-S randomized study. Eur Heart J Cardiovasc Pharmacother, 10:190-200.

[5] George M, Jena A, Karthikeyan BJIjob, pharmacology c (2016). Cangrelor - rising from the ashes: a phoenix story. 5:225-228.

[6] Ries T, Siemonsen S, Grzyska U, Zeumer H, Fiehler J (2009). Abciximab is a safe rescue therapy in thromboembolic events complicating cerebral aneurysm coil embolization: single center experience in 42 cases and review of the literature. Stroke, 40:1750-1757.

[7] Giordano A, D'Angelillo A, Romano S, D'Arrigo P, Corcione N, Bisogni R*, et al.* (2014). Tirofiban induces VEGF production and stimulates migration and proliferation of endothelial cells. Vascul Pharmacol, 61:63-71.

[8] Hasdai D, Holmes DR, Jr., Criger DA, Topol EJ, Califf RM, Wilcox RG*, et al.* (2000). Cigarette smoking status and outcome among patients with acute coronary syndromes without persistent ST-segment elevation: effect of inhibition of platelet glycoprotein IIb/IIIa with eptifibatide. The PURSUIT trial investigators. Am Heart J, 139:454-460.

[9] Weyrich AS, Denis MM, Kuhlmann-Eyre JR, Spencer ED, Dixon DA, Marathe GK*, et al.* (2005). Dipyridamole selectively inhibits inflammatory gene expression in platelet-monocyte aggregates. Circulation, 111:633-642.
[10] Schrör K (2002). The pharmacology of cilostazol. Diabetes Obes Metab, 4 Suppl 2:S14-19.
[11] Lesault PF, Boyer L, Pelle G, Covali-Noroc A, Rideau D, Akakpo S*, et al.* (2011). Daily administration of the TP receptor antagonist terutroban improved endothelial function in high-cardiovascular-risk patients with atherosclerosis. Br J Clin Pharmacol, 71:844-851.
[12] Lam S, Tran T (2015). Vorapaxar: A Protease-Activated Receptor Antagonist for the Prevention of Thrombotic Events. Cardiol Rev, 23:261-267.
[13] Rosano GMC, Spoletini I, Gianni W, Vitale C (2018). New Advances in Atrial Fibrillation Management: The Role of Apixaban. Curr Drug Targets, 19:585-592.
[14] Ahmed S, Levin V, Malacoff R, Martinez MW (2012). Dabigatran: a new chapter in anticoagulation. Cardiovasc Hematol Agents Med Chem, 10:116-123.
[15] Spencer RJ, Amerena JV (2015). Rivaroxaban in the Prevention of Stroke and Systemic Embolism in Patients with Non-Valvular Atrial Fibrillation: Clinical Implications of the ROCKET AF Trial and Its Subanalyses. Am J Cardiovasc Drugs, 15:395-401.
[16] Agnelli G, Buller HR, Cohen A, Curto M, Gallus AS, Johnson M*, et al.* (2013). Apixaban for extended treatment of venous thromboembolism. N Engl J Med, 368:699-708.
[17] Corbalán R, Nicolau JC, López-Sendon J, Garcia-Castillo A, Botero R, Sotomora G*, et al.* (2018). Edoxaban Versus Warfarin in Latin American Patients With Atrial Fibrillation: The ENGAGE AF-TIMI 48 Trial. J Am Coll Cardiol, 72:1466-1475.
[18] Hirsh J, O'Donnell M, Eikelboom JW (2007). Beyond unfractionated heparin and warfarin: current and future advances. Circulation, 116:552-560.
[19] Christiansen HM, Borris LC, Lassen MR (1989). [Low-molecular weight heparin. A new agent to prevent thrombosis]. Ugeskr Laeger, 151:1302-1305.
[20] Giangrande PL (2002). Fondaparinux (Arixtra): a new anticoagulant. Int J Clin Pract, 56:615-617.
[21] Piriou PG, Manigold T, Letocart V, Guérin P, Vourc'h M (2023). BRIGHT-4 trial: bivalirudin strikes back. Lancet, 401:1157-1158.
[22] Kondo LM, Wittkowsky AK, Wiggins BS (2001). Argatroban for prevention and treatment of thromboembolism in heparin-induced thrombocytopenia. Ann Pharmacother, 35:440-451.
[23] de Pont AC, Hofstra JJ, Pik DR, Meijers JC, Schultz MJ (2007). Pharmacokinetics and pharmacodynamics of danaparoid during continuous venovenous hemofiltration: a pilot study. Crit Care, 11:R102.
[24] Dobesh PP, Trevarrow BJ (2019). Betrixaban: Safely Reducing Venous Thromboembolic Events with Extended Prophylaxis. Am J Med, 132:307-311.

© 2025. Zhang W et al. Published online at http://www.aginganddisease.org/EN/10.14336/AD.2024.1668
